# The ParentingWell Practice Approach: Adaptation of Let’s Talk About Children for Parents With Mental Illness in Adult Mental Health Services in the United States

**DOI:** 10.3389/fpsyt.2022.801065

**Published:** 2022-04-07

**Authors:** Joanne Nicholson, Miriam Heyman, Kelly English, Kathleen Biebel

**Affiliations:** ^1^The Heller School for Social Policy and Management, Institute for Behavioral Health, Brandeis University, Waltham, MA, United States; ^2^The Heller School for Social Policy and Management, Lurie Institute for Disability Policy, Brandeis University, Waltham, MA, United States; ^3^Children, Youth and Family Services, Massachusetts Department of Mental Health, Boston, MA, United States; ^4^Massachusetts Rehabilitation Commission, Boston, MA, United States

**Keywords:** parents with mental illness, adult mental health services, intervention adaptation, family-focused practice, recovery

## Abstract

**Background:**

Despite the importance of family and parent-focused practice, there has been a dearth of research on interventions for parents with mental illness. This paper describes the process and outcome of adapting an evidence-based intervention, Let’s Talk about Children (LTC), in the context of adult mental health services in Massachusetts, United States.

**Methods:**

Specific objectives included: (1) to specify the core components, functions, and principles of LTC essential to adapting the intervention (i.e., program theory), (2) to consider contextual factors related to the new setting; (3) to pre-test the adapted materials with diverse practitioners; and (4) to compile the program model and materials (i.e., the practice profile) for use by adult mental health service providers in Massachusetts. The Adaptation Team included individuals with expertise in psychiatric rehabilitation and clinical care, policymaking, program development and research, and parents. Activities occurred between 2015–2019 and included: (1) consulting with experts to specify the core elements and theory behind the selected intervention (i.e., with the LTC purveyor and international experts); (2) consulting with key stakeholders for input regarding the Massachusetts target population and context to inform adaptations (i.e., individual and group key informant interview sessions); (3) pretesting the initial adapted materials (i.e., training and coaching sessions with adult mental health practitioners); and (4) using feedback to refine and compile the final intervention manual (i.e., the ParentingWell Practice Profile). Participants reflected diverse, oftentimes multiple roles and perspectives, including those of parents with mental illness, adult children, and family members.

**Results:**

ParentingWell is practitioner- and setting-agnostic, addresses parenting across the lifespan, fits into the routine workflow, and builds on practitioners’ existing skills. Eight themes emerged, which were translated into four core elements (engage, explore, plan, access and advocate) consistent with Self-Determination Theory and four underlying principles (trauma-informed, strengths-based, family-focused, culturally sensitive) in keeping with the LTC model. The ParentingWell Practice Profile operationalizes each core element and addresses the underlying principles.

**Conclusion:**

ParentingWell makes talking about parenting and family experiences a routine part of the therapeutic conversation with adults with mental illness. Future research will test the adaptation, implementation, and impact of ParentingWell.

## Introduction

Family-focused practice has received increasing attention over the past decade, particularly in relation to parents with mental illness receiving care in adult mental health services (1–8). The relative lack of research into interventions for families with parents with serious mental illness has been highlighted (9). Practitioners have reported or been found to have significant deficits in relevant skills, knowledge and confidence in working with adults who are parents and their families (4, 5, 7–12). Challenges in integrating family-focused interventions into everyday routine in adult mental health are context- as well as practitioner-related (13). Contextual issues that may impede adoption or result in the adaptation of specific interventions include perceptions of workplace support (12); the need for training, mentoring, supervision and co-worker support (5); the fact that the implementation of new routines is a time and resource consuming effort (14); and the challenge of taking an open approach to the definition of family—a “whole of family” approach—in a context that is focused on the assessment and treatment of the individual (1, 6). We undertook the task of addressing these issues as we navigated the process of identifying and adapting an evidence-based intervention, Let’s Talk about Children, to meet the needs of adults with serious mental illness who are parents or planning to become parents, in the context of adult mental health services in Massachusetts, United States.

Extensive groundwork for the selection and adaptation of an appropriate evidence-based practice was laid in previous research on the prevalence, experiences and needs of parents living with mental illness (15, 16), prior assessment of community capacity and needs (10, 17), and the prior identification and evaluation of existing models (18–22). Prior work was conducted in partnership with parents, practitioners, and policymakers, in the spirit of participatory action research and the mantra “nothing about us without us.” We reviewed existing evidence-based interventions, selecting Let’s Talk about Children (LTC), a three session, well-articulated, prescribed model developed in Finland (15, 23–26) and replicated and tested in Australia (13, 27–29), Greece (30), and Japan (31). The research background and replication of LTC in different countries were described in detail in a recent paper published in the current Frontiers special topic collection (32).

The goal of LTC is to promote parenting and child development and prevent children’s mental health problems by providing their parents information and opportunity to talk about their children. The provider is trained to use a semi-structured interview tool in three or four prescribed sessions to guide the discussion about parenting to address the child’s life, the parent’s mental illness and its meaning for the family, development of a plan to promote the child’s wellbeing and family life, and the engagement of supports and services. It is important to note that in randomized controlled trials in countries other than Finland, adaptations have been made to enable engagement with the parent (e.g., changing language to fit a parent’s needs or culture), to fit the service system or model of care (e.g., delivering LTC in shorter sessions over a longer period of time), and to tailor LTC materials (e.g., to incorporate changes in questions asked) (13, 32).

In addition to specific guidelines for implementing the LTC model, authors have recommended the importance of six core and inter-related principles of family-focused practice for families living with parental mental illness that informed our work including: (1) family care planning and goal setting; (2) liaison between families and services including family advocacy; (3) instrumental, emotional and social support; (4) assessment of family members and family functioning; (5) psychoeducation; and (6) a coordinated system of care (1). Others stress the benefit of assessing strengths within families, a non-judgmental and supportive approach, transparency to build trust, and the normalization of parenting difficulties (8, 33). International efforts to extract and replicate key elements of family-focused practice and develop program theory have recently been described, with a focus on consideration of the relationships among contextual factors, action mechanisms, and impact (34).

The tension between intervention fidelity and fit as evidence-based practices are implemented in real world settings has given rise to the science of adaptation (35). In the context of innovation diffusion theory, Rogers defined adaptation as “the degree to which an innovation is changed or modified by a user in the process of its adoption and implementation” (36). Adaptation has come to represent a more active, intentional process “to make an intervention fit a specific or new use or situation often by modification” (37). Adaptation of interventions, developed and tested in more controlled conditions, has been suggested as a requirement for achieving sustainable real-world outcomes, attending to intervention fidelity while adjusting to local needs and contingencies operating in the environment (38). Aarons and colleagues further specify types of “scaling out,” when evidence-based practices are adapted to new populations or new delivery systems or both (37, 39). Strategic, well-considered implementation of an evidence-based practice with a different population or in a different setting may contribute to more expeditious testing in a shorter timeframe. Strength may be “borrowed” from evidence obtained in prior effectiveness trials to the extent that core elements or core functions and forms are retained (39, 40).

Authors consistently highlight the importance of a systematic approach, both to navigating the adaptation process as well as documenting the adaptations made (35, 37, 39, 41, 42). Prior studies of intervention adaptation have focused on public health interventions including HIV prevention, teen pregnancy, and sexually transmitted infection (41). The recommendation has been made that more studies of the adaptation process to improve fit between interventions and contexts would inform adaptation strategies in the context of implementation (35).

Several models of the steps or phases in the adaptation process have been outlined (36, 37, 40–44). Common among them are steps involving exploration, preparation, implementation, and sustainment, with similar accompanying activities. Authors acknowledge that the adaptation process is not necessarily linear, but is best described as an iterative, dynamic process in which steps may overlap, with feedback loops informing next steps and refinements (36). At best, key stakeholders are engaged throughout the process, to ensure that all interests are represented, that the adapted intervention is culturally sensitive and relevant, and to promote stakeholder buy-in, thereby increasing the likelihood of successful implementation and positive outcomes (41). The ultimate goal of the adaptation process is to maintain as much fidelity to the essential ingredients of the original model as possible, while facilitating fit and feasibility with the new target population or context (42).

A multidisciplinary Adaptation Team, including researchers, practitioners, implementers, and service recipients or consumers, is recommended to guide and navigate this process (35). The first phase, exploration, generally involves assessing needs, selecting an intervention, and gathering and reviewing relevant intervention or program model materials. If possible, the developer or purveyor, and other experts are involved to ensure the Adaptation Team fully understands the selected intervention and the context in which it was originally implemented. Core elements or components (i.e., key ingredients necessary to make the intervention effective), core functions and forms (i.e., intervention activities that produce change) or best practice characteristics (i.e., characteristics common to effective programs) of the original model are identified, along with the internal logic or theory of change (36, 39–41, 44).

The second phase of the adaptation process focuses on the preparation of the adapted intervention or program model and materials (44). Common activities include the identification of mismatches between the original intervention or program model and the new context (e.g., culture, health care system, social and economic disparities), to enhance the potential fit and feasibility of the adapted model. This task may be informed by interviews with key community stakeholders to promote understanding of the contextual differences. Description of the adapted intervention model and materials (e.g., manual, training resources) may be reviewed by community partners and representatives of the target population, and feedback solicited (44).

The activities of the implementation phase generally involve the pre- or pilot testing of the adapted model, with training of staff, taking model adaptations into account. Intervention components and procedures are evaluated and refined (44). Finally, in the sustainment step, the adapted intervention is implemented and evaluated further, training and supervision provided on an ongoing basis, and a dissemination plan implemented. Attention to issues of training and ongoing technical assistance promote better intervention results (38).

The purpose of this paper is to describe the process of adapting an evidence-based intervention, Let’s Talk about Children (LTC), targeting parents with mental illness receiving services in the adult mental health system in Massachusetts. The specific objectives included: (1) to explore and specify the core components, functions, and principles of LTC essential to adapting the intervention (i.e., program theory), (2) to consider contextual factors related to the new setting (i.e., practice, organizational and systemic factors); (3) to pre-test the adapted materials with diverse practitioners working with parents (i.e., in training and coaching sessions); and (4) to compile the program model and materials (i.e., the practice profile) for use by adult mental health service providers in Massachusetts. The overall project goal was to adapt LTC and clearly specify a program model for parents with mental illness that could be implemented, tested and sustained in the context of adult mental health services. We partnered with diverse stakeholders including parents with mental illness, their children, and family members to specify and adapt an appropriate model, pre-test, and refine the model for scale-up and future, larger-scale implementation, rigorous testing, and sustainment.

## Materials and Methods

A developmental evaluation design and qualitative methods provided the framework for the iterative process of exploration and innovation in adapting the LTC model (45). Cycles of data collection, reflecting, feedback and refinement were not linear, as adaptation activities informed each other in a reflective manner and changes were made based on emergent conditions and information. Consequently, findings from multiple perspectives were integrated systematically over time to inform the final program model and practice profile.

### Procedures

Adaptation activities occurred between 2015–2019 and included: (1) consulting with experts with professional and lived experience to explore and specify the core elements and theory behind the selected intervention (i.e., with the LTC purveyor and international experts, in individual and group in-person and videoconference sessions); (2) consulting with key stakeholders with professional and lived experience for input regarding the Massachusetts target population and context to inform program model adaptations (i.e., individual and group stakeholder interview sessions); (3) pretesting the initial adapted materials (i.e., training and coaching sessions with adult mental health practitioners working with parents); and (4) using feedback to make further modifications and compile the final intervention manual (i.e., the ParentingWell Practice Profile). These steps were not completely linear, in that an iterative process of considering adaptations and checking back with stakeholders occurred over time, consistent with a developmental evaluation design. A core Adaptation Team met over time to facilitate the adaptation process. It is important to note that, while stakeholders may be referred to as “practitioners,” for example, practitioners may reflect diverse perspectives and multiple roles and responsibilities (e.g., peer specialists who themselves are parents with mental illness).

Procedures for each phase of the project were reviewed by the relevant university and state agency institutional review boards. When activities met criteria for human subjects research *per se*, the appropriate written or verbal consents were obtained, as recommended by the institutional review boards. Stakeholders (i.e., agency staff, policymakers, parents, adult children, family members, and advocates) were volunteers who did not receive stipends for participation, as all activities took place during routine working hours as part of ongoing professional and agency activities and commitments.

#### The Adaptation Team

A core group served as the Adaptation Team (*n* = 4), including parents and individuals with backgrounds and expertise in psychiatric rehabilitation and clinical care, policymaking, program development and research. The Adaptation Team met in person, bi-weekly, and communicated more frequently via email and text message throughout the 4 years of the project. Detailed minutes were typed directly into electronic documents for qualitative analysis and stored in secure digital files by independent research staff who observed the meetings.

#### The Let’s Talk About Children Purveyor and International Expert Group

The Let’s Talk about Children (LTC) purveyor (Solantaus) and colleagues met quarterly in 2-h sessions over the course of 2 years (2015–2017) via video conferencing as the LTC Worldwide Group (*n* = 20). The group included purveyors, researchers and practitioners from Finland, Japan, Australia, Sweden, Italy and the United States, with professional and lived experience, who discussed implementation issues in diverse practice settings internationally. Adaptation Team members presented draft materials and Massachusetts-specific implementation considerations for input and feedback from LTC Worldwide members. In addition, individual LTC Worldwide participants provided in-depth review of draft project materials and detailed feedback. Meeting presentations, detailed minutes and reviewers’ comments were transcribed into electronic documents by research staff and stored in secure digital files.

#### Key Massachusetts Stakeholders

Twelve individual or group interview sessions of approximately 1 to 2 h each were completed early in the project (2016) by telephone or in-person, involving a convenience sample of 70 participants with professional and lived experience recruited by telephone and email to represent the Massachusetts Department of Mental Health leadership and Planning Council, advocates from the National Alliance of Mental Illness, and two community-based agencies providing outpatient and residential mental health services to adults, and parents themselves. The agencies provide diverse mental healthcare services to 40,000–50,000 individuals and families annually with sites located in diverse geographic areas in Massachusetts. Agency representatives included practitioners (i.e., peer specialists, clinicians, case managers), supervisors, program managers and agency administrators, who participated in invited staff gatherings. Members of the Adaptation Team provided informational and draft materials for participants to review and facilitated discussion regarding topics including: (1) the experiences of parents and practitioners; (2) services currently provided; (3) challenges and unmet needs; and (4) implementation issues, current or anticipated, related to the agency and community contexts. In-person interviews took place in comfortable agency settings (e.g., a large office or conference room). Detailed verbatim notes were compiled electronically and stored in secure digital files by an independent research staff member attending meetings and using a laptop computer. A draft manual was compiled, based on the input and feedback of stakeholders to this point.

#### Adult Mental Health Service Practitioners

Two, 2-h in-person training sessions, held 2 weeks apart in May 2017, were conducted by members of the Adaptation Team in each of the two community-based agencies providing mental health services to adults. Practitioners were provided with the draft manual, and training content focused on information and materials in the draft manual to pretest materials. Twelve practitioners (e.g., social workers, peer specialists, case managers, supervisors) working with parents with mental illness participated. Participants then attended 41-to-2-h in-person coaching sessions, held in each agency at 1-month intervals following the training, in a comfortable conference room, facilitated by the Adaptation Team members. Participants were encouraged to describe contacts with parents served and supported in sharing suggestions for strategies to deal with challenges in service provision. Again, detailed notes were entered into document files by independent research staff members using laptop computers; documents were then stored in secure digital files.

### Analysis

The goal of the project was to describe the process of intervention adaptation and compile the refined intervention model, rather than assess the impact of an intervention on practitioner or parent outcomes. Consequently, detailed background and demographic data on individuals participating were not solicited. Participants represented multiple roles and responsibilities, professional and lived experience, and their “in the moment” contributions reflected any of these. Detailed notes, systematically obtained and recorded in many diverse settings from multiple perspectives over time, were captured via laptop or transcribed where necessary into electronic documents by independent research staff, uploaded into Dedoose software to facilitate data management and coding, and reviewed systematically by members of the research team (46). The Adaptation Team and research staff debriefed after each consultation, interview, training or coaching session to review key points, add to notes as necessary to insure the thoroughness of documentation, and plan for the next data collection opportunity. Themes were identified and elaborated in content analysis of transcribed documents, across participants’ perspectives, by members of the research team, experienced qualitative researchers, who met regularly to develop a shared understanding of themes and related codes. Transcripts were coded independently by members of the research staff who met to discuss and reconcile any differences, and to inform and refine subsequent coding. Themes identified through review and coding of prior sessions were explored further in subsequent sessions with diverse participants, to corroborate and elaborate data and thematic codes, and to obtain input and feedback from multiple perspectives over time (i.e., triangulation and member checking) (47). Memos were generated by research staff, describing and elaborating themes across data sources, to facilitate the identification of patterns and relationships among themes. These memos were reviewed by research staff and Adaptation Team members with professional and lived experience for further elaboration and feedback until consensus was achieved on key findings. Findings were compiled and translated into the final ParentingWell Practice Profile, to operationalize program theory and key intervention components, and provide guidelines for practitioners’ interactions with parents served.

## Results

Findings relate to the study goal of adapting the LTC intervention to the new context and service setting. Named “ParentingWell,” the core elements of LTC are retained, while shaping the practice approach to fit the service context and practitioners’ recommendations. Concrete strategies for intervening are elaborated, based on the core elements and practice principles derived from LTC and translated for application in the U.S. setting.

### Specifying the ParentingWell Program Theory

**FIGURE 1:**
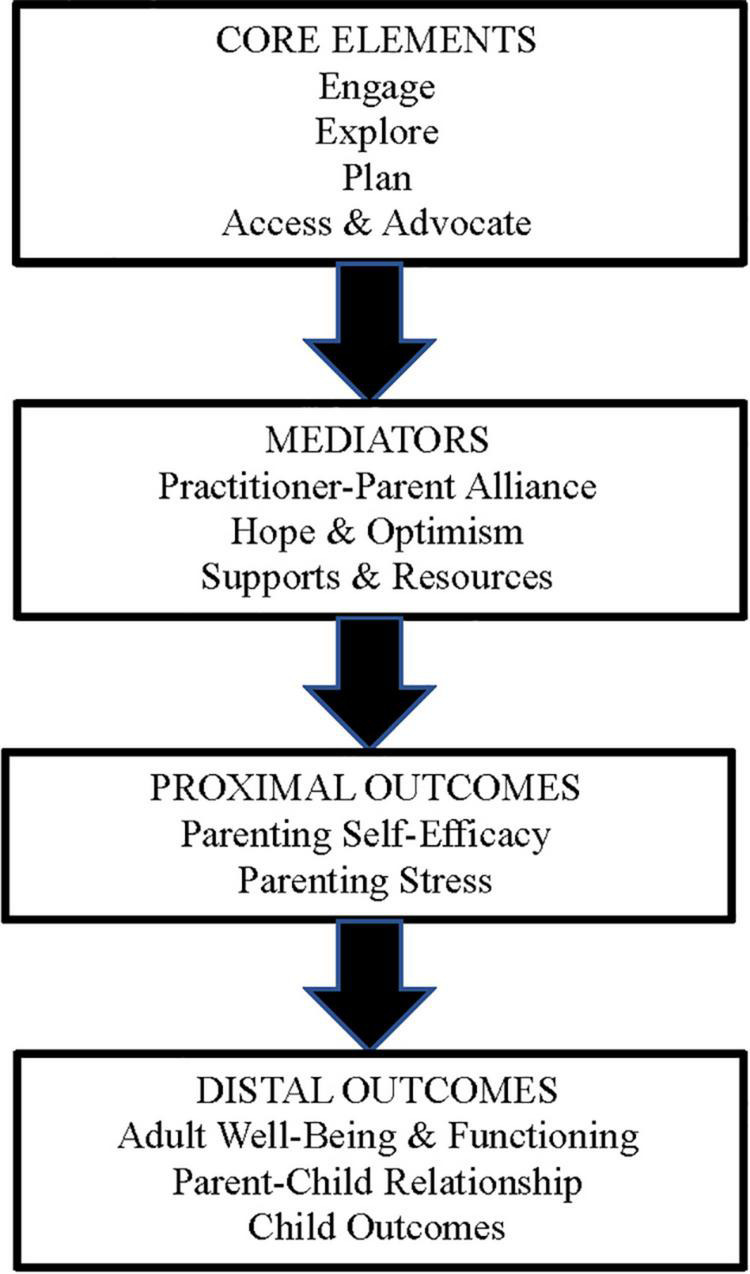
The ParentingWell program theory is based on Self-Determination Theory (46) with core elements of Engage and Explore (autonomy: identifying personal circumstances and motivation, to feel and do better. Plan (competence: setting goals, assessing progress, and building self-efficacy), and Access and Advocate (relatedness: linking to natural and professional supports and resources).

The ParentingWell program theory or logic model, adapted from the key elements of the Let’s Talk about Children intervention, is based on Self-Determination Theory (48) with core elements of Engage and Explore (*autonomy*: identifying personal circumstances and motivation), Plan (*competence*: setting goals, assessing progress, and building self-efficacy), and Access and Advocate (*relatedness*: linking to social and professional supports and resources). Recent work by Australian colleagues corroborates the consistency of LTC underpinnings with Self-Determination Theory, that is, of the need to feel autonomous, effective and connected as drivers of the mental health recovery of parents (49). The original LTC model was found to enable practitioners to support parents with mental illness in building agency and self-regulation (49). We hypothesize practitioner outcomes to include enhanced skill and comfort, increased use of ParentingWell resources, and more frequent interactions with adults regarding parenting and family life, strengths, goals and needs, which will contribute to the model mediators of practitioner-parent alliance, hope and optimism, and supports and resources. We hypothesize proximal parent outcomes to include enhanced parent self-efficacy and reduced parenting stress. Distal outcomes include improved adult/parent wellbeing and functioning which will, in turn, contribute to and benefit from improvements in the parent-child relationship and enhanced child outcomes (see Figure 1).

### Considering the New Context

We found numerous challenges to the implementation and spread of the adapted ParentingWell model when closely replicating the three-session LTC model. We learned that what practitioners needed and wanted, rather than a tightly prescribed intervention protocol, was a more loosely described practice approach, with well-specified principles and core elements, that fit into their routine work flow, drew from the skills and competencies they already had, was perceived as enhancing their work and promoting the recovery of the people with whom they worked, and could be of value in working with the parent of children at any age, at any point in the therapeutic relationship. In addition, work with parents and the spread of ParentingWell practice was perceived to benefit from training, coaching and ongoing support, professionally and organizationally.

Specific points of feedback included the following: (1) practitioners should routinely ask about parenting and family life; (2) parenting and family life must be integrated into program staff and agencies’ routine practice, and not isolated as a specialty service; (3) working together with parents is complex and practitioners would benefit from targeted, ongoing support; (4) shifting to a focus on parenting and family life with adult providers requires organizational champions and support; and (5) organizations must be prepared to adapt policies, practices, and the agency context to be parent- and family-friendly. The first and second points both broadly relate to consistent integration of parenting considerations, as opposed to the implementation of an isolated intervention. In illustration of this need for consistent integration of parenting conversations into routine practice, one practitioner (coaching session, July 2017) noted the need to “link the parenting goals to other goals including symptom management, community education, community inclusion.” Another practitioner said (coaching session, November 2017), “Making things general, like symptom management, or adult daily living skills or housing or self-care—all these things can work back around to parenting (e.g., you need to do self-care so you can be able to care for your son),” clearly conveying synergies between parenting and other topics. A coaching participant expressed that she was hesitant to tack on an “additional component” to her routine, and thus supported the idea of more holistic integration into practice (coaching session, June 2017).

The third, fourth, and fifth points of feedback each depict the need to address organizational considerations to adequately support parents. These organizational considerations include support for practitioners who do this work, having organizational infrastructure and champions to ensure the consistency of the work, and having family-friendly policies within the agency. For example, one stakeholder mentioned “coaching and supervision” as key considerations for peer specialists (Adaptation Team session, December 2016). Another practitioner commented that “In our [comprehensive assessment] we have a risk assessment and different addendums—this could be an addendum—other ones are substance abuse history, medications, legal issues. We use these to develop treatment plans (key informant session, August 2016).” This practitioner thus conveyed the need to infuse parenting questions into other agency routines—a task related to organizational infrastructure. Suggested family-friendly policies included allowing toys in the waiting room and permitting parents to bring their children in vehicles when transportation was provided by the agency.

Our notion of success thus loosened to focus less on the specific details of ParentingWell as a prescribed intervention, and more on translating the underlying goals and principles into concrete practice recommendations and skills, and building the capacity of practitioner/adopters to adapt the ParentingWell practice approach in their own settings (50). As such, the adapted practice approach ultimately focuses on the following three questions, which should be woven into routine interactions between behavioral health providers and their clients: (1) What are your parenting and family circumstances? (2) How are things going? and (3) How would you like them to be?

### Pre-testing the ParentingWell Practice Approach

Eight themes (each described below) emerged from conversations with stakeholders and practitioners. These themes were ultimately translated into the core elements and underlying principles of the ParentingWell model, as described in a subsequent section.

Stakeholders consistently noted the need for a **family-focused** approach to behavioral health across the lifespan. As an LTC Purveyor explained, “Being able to respond to the family and child feeds into the sense of agency, which is a key ingredient in resilience” (LTC-Worldwide session, September 2015). Another stakeholder alluded to the role of the family in recovery, and the implications for behavioral health practice, as she said, “A lot of the clients that are parents do not have custody of their children and do not have visitation. We want these clients involved, because we feel issues of family life and children are incredibly important to recovery” (key informant session, October 2016). This stakeholder thus conveyed that a family-focused approach is relevant for the unique experiences of each client, including issues of custody loss and/or visitation, where relevant, and for those with children of any age.

Stakeholders acknowledged the fact that culture is largely influential in parenting and mental health (i.e., there is “diversity and cultural competency and different attitudes about parenting”; key informant session, June 2016), and thus recognized that the approach should be **culturally sensitive.** In discussing how to adapt existing models, one stakeholder stated that it will be important to “develop, when necessary, informed cultural adaptations in the Let’s Talk model without sacrificing its principles” (LTC-Worldwide session, September 2015). These adaptations may occur in the context of individual practitioner-client relationships, so that practitioners can explore the implications of clients’ cultures for their parenting experiences, family life and recovery.

Conversations with stakeholders frequently reflected their inclination to focus on the strengths of parents served, to inspire hope and to capitalize strengths to facilitate goal achievement. One practitioner reflected, “She (the mother) finds the conversations helpful in realizing the abilities she has within herself in helping her child and improving her parenting” (coaching session, September 2017). Thus, the **strengths-based** approach of these conversations was integral for this client in enabling her to focus on and grow positive aspects of her parenting experience. Another practitioner offered the following question that would be helpful to use within the ParentingWell approach: “Would you be interested in talking about your strengths and goals around parenting?” (key informant session, September 2016).

Stakeholders were also aware of the fact that many of their clients had experienced trauma, in some cases related to parenting, and thus the approach should be **trauma-informed**. A trauma informed approach would recognize the reality that “sometimes people are concerned that these types of questions [about parenting] will upset or retraumatize their clients” (key informant session, August 2016), especially for clients who do not have contact with their children. An Adaptation Team member (practitioner) noted that the approach should be trauma-informed, meaning that it needed “dependable, reliable, follow-through, non-judgmental” (Adaptation Team session, April 2018). The non-judgmental element is especially relevant given the wide array of parenting experiences, including the experience of separation from children.

Many stakeholders raised considerations regarding ways to **engage** with parents about family life respectfully and non-judgmentally and doing so in a way that aligns with the parents’ needs and preferences. Stakeholders raised concerns associated with engagement, as reflected in the following quote: “Sometimes people are concerned that these types of questions will upset or retraumatize their clients—that is an issue” (key informant session, August 2016). Despite these hesitations, stakeholders acknowledged the importance of engaging their clients in these potentially difficult conversations. As one stakeholder said, “Part of the wellness role is to validate experiences, including parenting ones” (key informant session, June 2017) Given the recognized need to include conversations about parenting in their interactions with clients, stakeholders shared strategies for doing so, including meeting the parents where they are at, and bringing up parenting when the parent seems ready and willing to do so, pacing the conversations. Stakeholders emphasized that listening to the parent is key: “Listen. Listen to what’s going on. Lots of people think they know what’s going on, but you really need to listen. Don’t be directive. Be collaborative in the way you work with someone. When people are directive, it pushes people away and people can get angry” (coaching session, August 2017).

Stakeholders also reflected on how to **explore** the wide range of their clients’ parenting experiences, some of which may be emotional experiences that are charged with shame and self-blame. One practitioner explained, “Some clients are afraid to even talk with their children about their diagnoses—a lot of the time children don’t even know what their parents are dealing with” (key informant session, August 2016). Stakeholders conveyed the importance of discussing family-related transitions and associated stressors: “Relating to how scary the leap of faith is as a parent when having their child moving in with them” (coaching session, September 2017) Another stakeholder conveyed the wide range of parenting experiences, and their associated implications for conversations about parenting, as she said, “We need to also realize that there are some parents with adult children… some parents want to make a connection with their older child” (key informant session, February 2016). Another stakeholder simply noted, “There are many ways to be a parent” (training session, May 2017).

Additionally, stakeholders explained that behavioral health practitioners should help parents in making plans to improve their experiences related to parenting. Thus, helping parents **plan** should be a key element of an approach to parenting-focused behavioral health approach. Parents will ultimately be encouraged to weave parenting goals into their overall wellness and recovery plan. As one participant said, “Feels like you could have a ParentingWell conversation about what was positive re: parenting and use that to start thinking about a plan. Focusing on how things are going, and how you’d like things to be” (coaching session, August 2017).

Stakeholders clearly conveyed that part of their role was to help their clients **advocate and access** peer supports, opportunities for self-care, supports related to basic living needs, and culturally relevant resources. Demonstrative quotes include: “Peer specialists are the ones who relate to the family, who the client will listen to. From the perspective of the peer specialist—‘I understand your situation. What would work for you? Who will be in your life? Who will be there to support you?” (key informant session, March 2016); and regarding what training or preparation workers would need: “…to help the client find home or shelter, things the baby would need, parenting classes, the social welfare benefits process, information about what is changing in the system, employment and benefits applications, Mass Health (health care payer) applications” (key informant session, March 2016).

### Compiling the ParentingWell Practice Profile

In light of the feedback we received, we shifted our focus to compiling the agency- and practitioner-agnostic ParentingWell Practice Profile (PWPP), relevant to parents across the lifespan (51). A practice profile describes the program or practice approach, including essential functions, operational definitions, and practical performance strategies (i.e., the theory of action). The PWPP provides concrete discussion points and topics (core activities) that practitioners can use to address the four core elements and four underlying principles. The core activities also embody action mechanisms (i.e., information sharing, reflecting and reframing, goal-setting, and skills-building; examples provided in the next paragraph). Thus, designed to reflect the core elements and underlying principles, and inclusive of concrete action mechanisms, the PWPP is the culmination of the adaptation process. The PWPP is also the operationalization of the core elements and practice principles into a specific theory of action (52) (see Table 1).

**TABLE 1:** The ParentingWell Practice Profile action mechanisms.

Information sharing	Respectful, non-judgmental curiosity
	Positive messaging (e.g., encouragement, empathy)
Reflecting	Exploring experiences, thoughts and feelings
and reframing	Understanding relationship between attitudes, thoughts and behavior Unraveling and challenging faulty thinking
	Recognizing patterns
	Taking the other’s point of view
	Shifting perspective to see a situation differently
Goal-setting	Forming intentions
	Identifying necessary resources (e.g., motivation, time and energy, natural, and professional supports)
	Pinpointing barriers and strategies for overcoming
	Setting SMART goals (Specific, Measurable, Achievable, Relevant, Time- Bound)
	Celebrating successes
Skills-building	Observing and recording (e.g., journaling) Instructing
	Modeling or demonstrating the behavior Rehearsing and experimenting
	Providing relevant, appropriate feedback

*The ParentingWell theory of action, related activities and practitioner skills are informed by Social Cognitive and Cognitive Behavioral Theory and the Information-Motivation Behavioral Model.*

For example, a core activity suggests that during the first meeting, the practitioner welcomes the parents and asks initial questions about parenting and family status (core element **engage**, key principle **family focused**, action mechanism **information sharing**). A second core activity suggests that the practitioner support the parent in identifying strengths and resources, particularly as they relate to parenting/relationships with children and family life, social support, and self-care (**explore**, **strengths-based**, **reflecting and reframing**). A core activity pertaining to goal setting is to help the parents identify what they want to change and picture the outcomes; an activity pertaining to skills-building is to assist with a problem-solving approach if parents cannot “put the pieces in place” to take steps forward. Thus, the core activities provide concrete action steps that put into motion the core elements and underlying principles.

Core activities are not necessarily meant to occur in a particular order, activities from different core elements may occur simultaneously, and practitioners and clients may work back and forth among activities over time. For each client, a more complete picture of the person as a parent and their priorities for family life will emerge. Practitioners will be able to work with parents to help them weave their goals for parenting and family life into their vision for change and plans for the future.

In addition to compiling the ParentingWell Practice Profile as a guide for practitioners, the ParentingWell Workbook of activities for practitioners and parents is available, along with the ParentingWell Self-Assessment and Supervisory Tools for use by practitioners and their supervisors. These resources are available in Supplementary Material linked to this article.

## Discussion

This study describes the process and outcome of adapting an intervention for parents with mental illness, for implementation and sustainment in Massachusetts adult mental health service agencies. Specific objectives were as follows: (1) identify the core components and principles of the original LTC intervention; (2) consider service delivery contextual factors, which would be sustained during the adaptation process; (3) pre-test the adapted materials, resulting in the specification of new core components and principles; and (4) compile the practice profile, translating core components and principles into a theory of action and core activities.

Regarding the first study objective, conversations with stakeholders yielded the program theory or logic model for Let’s Talk About Children. The elements of the logic model comprise the core components and principles of the original intervention that were retained during the compilation of the adapted ParentingWell model. Adaptations that fail to retain the key elements of an intervention may reduce the effectiveness of that intervention (53). While the goal of adaptation is to improve the efficacy of an intervention for a new specified context, the assumption is that the original intervention remains intact enough for evidence of its effectiveness to remain relevant even in its adapted form (53). To ensure that the adaptation is fidelity consistent (and thus that evidence for its effectiveness “translates”), the identification and maintenance of key components of the original intervention are critical to the adaptation process (53). In examining the extent and impact of adaptation, other considerations relate to both process and outcome, such as whether modifications were planned/proactive or unplanned/reactive; who made the decisions; what is modified (e.g., components, delivery method, etc.); and factors that influenced decisions (e.g., improve fit, align with cultural values, norms or priorities) (53). The adaptation process described in this paper included a consistent focus on the key elements of Let’s Talk About Children, contributing to a strong likelihood that evidence for the effectiveness of Let’s Talk About Children will also apply to the ParentingWell Practice Approach. However, as future research explores implementation of the ParentingWell Practice Approach, it will be important to investigate how the original intervention and its adapted elements each contribute to its impact.

Conversations with stakeholders, including intervention purveyors, ParentingWell Training and Coaching Participants, reflected themes that addressed the second and third study objectives. Regarding contextual considerations (the second objective), the first theme that emerged from our data relates to workflow; specifically, stakeholders emphasized that the adaptation should ultimately result in a framework that can be consistently integrated into practice, rather than a stand-alone intervention. As such, the resultant ParentingWell is an approach to routine practice that makes talking about parenting, children, and family experiences a natural part of the conversation and of an adult’s recovery process. The ParentingWell approach thus addresses contextual considerations, namely by avoiding challenges that would accompany “tacking on” an additional intervention, which may require extensive time and training (3). Stakeholders who are familiar with adult mental health service agencies in Massachusetts emphasized the benefits of this routine integration. Future research should also investigate the extent to which the approach is relevant for agencies in other states and perhaps countries.

Also related to future implementation beyond Massachusetts, the use of the ParentingWell approach does not require extensive clinical, counseling or practice skill specific to addressing parenting. This may facilitate implementation in a wide variety of settings. Research in several contexts has established that practitioners often lack knowledge and skills related to addressing their clients’ parenting roles (4, 5, 7–12). The ParentingWell approach enables and encourages practitioners to draw from the skills they already possess, while keeping parenting in mind. As such, it does not require a vast set of skills that are specific to addressing parenting. Future research will need to explicitly address this characteristic as it relates to scale-up.

Regarding the third objective, stakeholders specified the following themes, which comprise the underlying principles and the core elements of the ParentingWell Practice Profile: the need for an approach that is family-focused, trauma-informed, culturally sensitive, and strengths-based; and for conversations in which the provider and the client engage, explore, plan, and access and advocate around issues related to parenting and family life. The identification of themes fulfills the third objective, which is the specification and the compilation of the ParentingWell Practice Profile. The Profile includes operationalized core activities for each element (i.e., for *engage*, the practitioner might ascertain where children are living and who is caring for them; for *explore*, the practitioner might discus daily routines, household chores, and taking care of the children). It also identifies how the underlying principles map onto each core element. For instance, *explore* is strengths-based as the activities reflect the understanding that parents, especially those who are quite depressed or see themselves as “failures,” may require assistance in identifying strengths and resources in themselves and in their children. Future research should assess the feasibility of implementing the approach, the impact on practitioner behavior, and ultimately, the impact on parents. Also, because the adaptation involved modifying a stand-alone intervention into a continuous and holistic approach, future research that assesses impact should seek to understand how this change impacts parents. Considerations might include whether the timing and/or duration of impact differs as a result of the transition from a stand-alone intervention to an ongoing approach.

The adapted model (the fourth objective) fills a critical gap as it addresses the lack of evidence-based interventions for parents with serious mental illness and it reflects the need for a flexible practice approach. The ParentingWell Practice Approach includes well-specified principles and core elements, aligned with core activities that constitute a theory of action. It fits into routine workflow at any point in the therapeutic relationship, and draws from practitioners’ existing skills and competencies, ultimately with the potential to enhance clients’ recovery.

### Limitations

Despite this promise, this study has its limitations. The stakeholders are reflective of the Massachusetts mental health workforce and, consequently, are mostly White. Meanwhile, both parenting and mental health are culturally bound. ParentingWell addresses this consideration, as a key principle is to be culturally sensitive, but it is still critical to engage more diverse stakeholders. This should be the focus of future testing and refinement of the ParentingWell Practice Profile. Additionally, the context of mental health service provision has changed with COVID-19, as has the context of parenting. The approach is designed to be flexible, delivered, however, and wherever mental health services are delivered. However, as future research explores the feasibility and impact of the approach, changing contextual factors should be kept in mind.

## Conclusion

Ultimately, this study and the adapted ParentingWell resources address the critical lack of evidence-based interventions for parents with serious mental illness. Future research will provide needed insight pertaining to its implementation and impact.

## Data Availability Statement

The datasets presented in this article are not readily available because given the qualitative nature of the data and the concern for the privacy of participants, many of whom are agency leaders, data are held by the authors. Requests to access the datasets should be directed to JN, jnicholson@brandeis.edu.

## Ethics Statement

The studies involving human participants were reviewed and approved by the Brandeis University Institutional Review Board, Dartmouth College Institutional Review Board, and Massachusetts Department of Mental Health Central Office Research Review Committee. The patients/participants provided their written informed consent to participate in this study.

## Author Contributions

JN was responsible for the overall design and implementation of study activities and the preparation of the manuscript. KE and KB contributed to study design and implementation. JN and KE were responsible for drafting the ParentingWell Practice Profile. MH contributed to data analysis and writing and editing of the manuscript. All authors contributed to the manuscript, read and approved the submitted version.

## Author Disclaimer

The statements presented in this publication are solely the responsibility of the authors and do not necessarily represent the views of the funders.

## Conflict of Interest

The authors declare that the research was conducted in the absence of any commercial or financial relationships that could be construed as a potential conflict of interest.

## Publisher’s Note

All claims expressed in this article are solely those of the authors and do not necessarily represent those of their affiliated organizations, or those of the publisher, the editors and the reviewers. Any product that may be evaluated in this article, or claim that may be made by its manufacturer, is not guaranteed or endorsed by the publisher.
