# Supplementary material for: The ParentingWell Practice Approach: Adaptation of Let’s Talk About Children for Parents With Mental Illness in Adult Mental Health Services in the United States
Source: Front Psychiatry. 2022 Apr 7;13:801065. doi: 10.3389/fpsyt.2022.801065 (PMC9021592; doi:10.3389/fpsyt.2022.801065)
Supplement: Supplementary file 1 [file Data_Sheet_1.PDF]

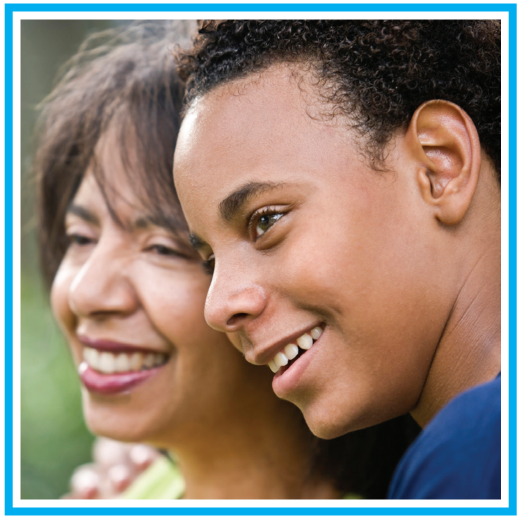
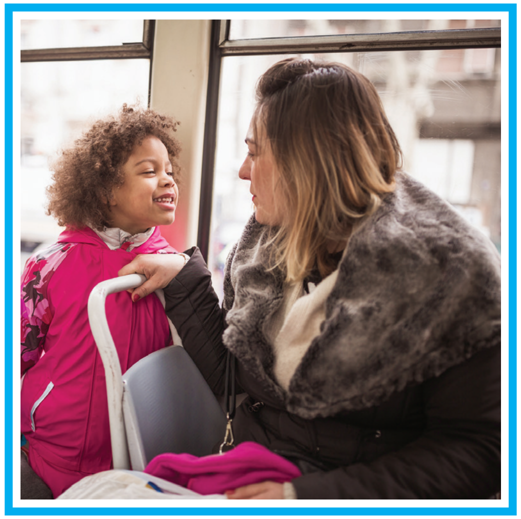
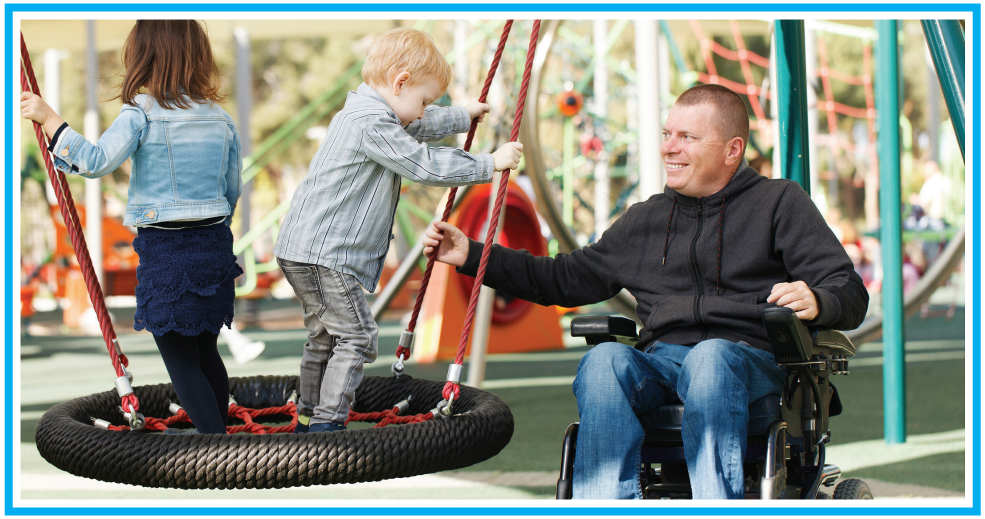
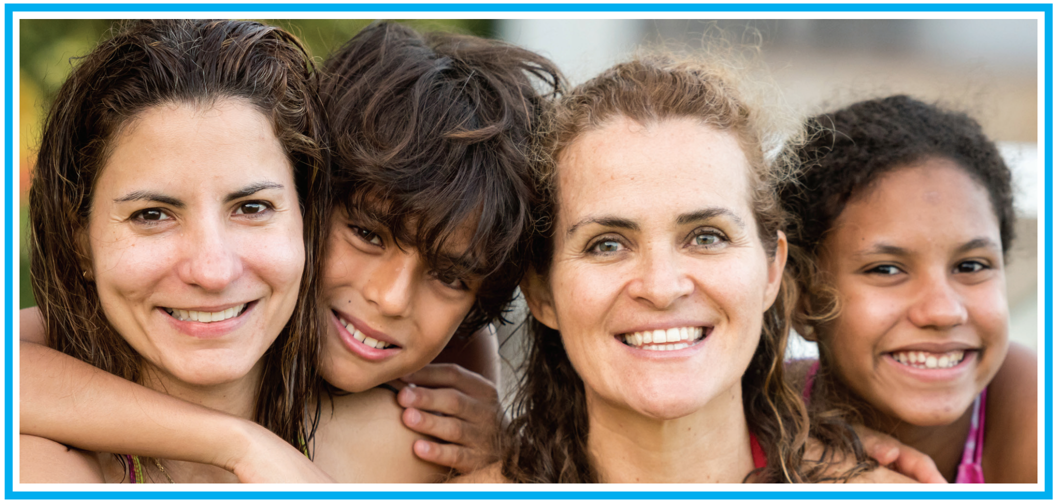

ParentingWell
PRACTICE PROFILE

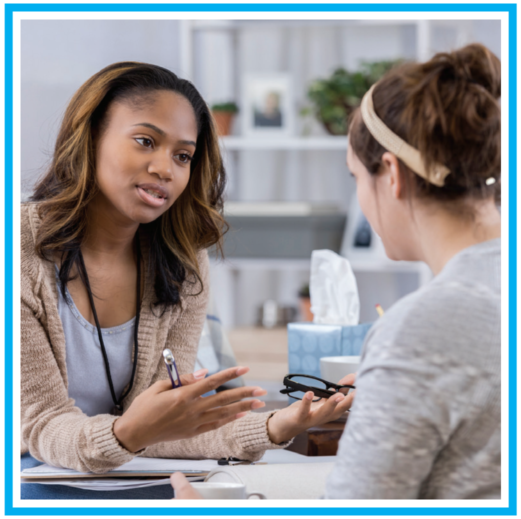
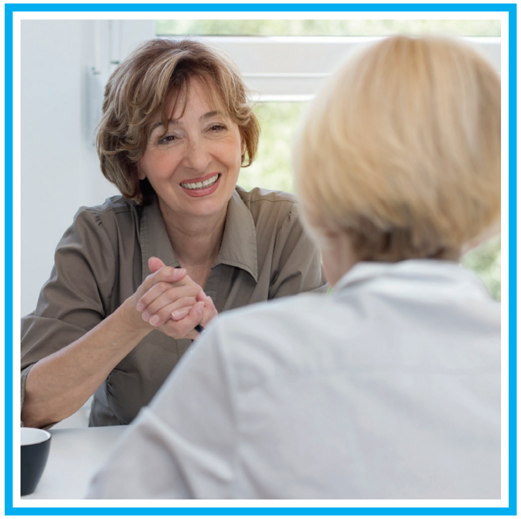
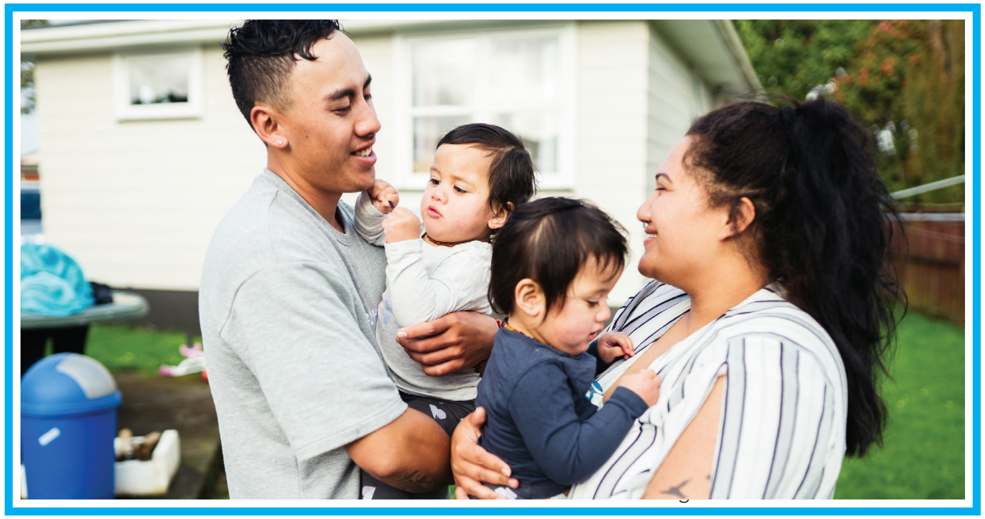

Acknowledgments

The ParentingWell® Practice Profile is funded by the Massachusetts Department of Mental Health and by a grant from the National Institute on Disability, Independent Living, and Rehabilitation Research[1] as part of the National Research Center on Parents with Disabilities at Brandeis University.

Researchers, policy makers, practitioners, advocates, parents, and families in Massachusetts have a long-standing commitment to the participation of individuals with serious mental illnesses in all aspects of community life, including family life and parenthood. Initial work on the ParentingWell practice approach began in the late 1990's with funding and support over the years from the Massachusetts Department of Mental Health (DMH), the National Institute on Disability and Rehabilitation Research[2], and the Substance Abuse and Mental Health Services Administration Center for Mental Health Services.

ParentingWell When You're Depressed (New Harbinger Publications, Inc., 2001), an earlier publication, was a joint effort of researchers, policy makers, and practitioners from the DMH, the Center for Mental Health Services Research at the University of Massachusetts Medical School, a consortium of clubhouses and members from across the Commonwealth, and the leadership and staff of Employment Options, Inc. in Marlborough, MA. The work has always been informed and conducted by individuals with lived experience of mental health and substance use conditions, their family members, and advocates.

Most recently, policy makers, program administrators, practitioners, advocates, parents with lived experience, and family members actively contributed to the work through focus groups, working retreat sessions, key informant interviews, implementation pilot studies, and the Parent Peer Specialist Implementation Team.

[1] (NIDILRR grant #90DPGE0001-01-01) The statements presented in this publication are solely the responsibility of the authors and do not necessarily represent the views of the federal funding agency.

[2] (NIDRR grant #H133G970079)

Citation: Nicholson J, English K. ParentingWell Practice Profile [Internet]. Waltham, MA: National Research Center for Parents with Disabilities, Brandeis University and the Massachusetts Department of Mental Health; 2019. Available from: https://heller.brandeis.edu/parents-with-disabilities/parenting-well/index.html

We are grateful to many contributing staff members from the DMH central office, regional and agency offices, and interns. Staff and consumers from Riverside Community Care, Eliot Community Human Services, Advocates, and Employment Options, Inc. worked with us to develop, review, and pilot the ParentingWell Practice Profile materials.

Key contributors from the DMH included Margaret Guyer-Deason, Robert Walker, Val Comerford, Beth Lucas, Sue Wing, and Joan Mikula. Christine Furnari from Vinfen Corporation served as an intern on the project.

Our colleagues Toni Wolf, Kathleen Biebel, and Chip Wilder have been involved in the family projects for several years and continue to inform and support the work. They were major contributors to *Creating Options for Family Recovery* (Employment Options, Inc., 2014), another stepping stone on the pathway to a family-focused approach to adults living with mental health conditions. Anne Valentine contributed as project manager at the Brandeis Parents' Center.

International colleagues have actively informed the development of the Practice Profile. The Let's Talk About Children model, developed by Tytti Solantaus and implemented in sites around the world, provided a starting point for adaptation and implementation in Massachusetts. Australian colleagues implementing a similar approach "down under," including Angela Obradavic, Rose Cuff, and Melinda Goodyear, reviewed prior drafts.

The current version of the ParentingWell Practice Profile is a collaborative effort compiled with the support of Kathryn Swaim, Tara Hall, and the resources of the UMASS Donahue Institute.

We are grateful to these individuals for their expertise, commitment, and persistence and for valuing the families with whom we collaborate. This Practice Profile is the result of our active partnership and diligent devotion to insuring that parenting and family life are possible options for all those who choose to pursue these goals.

Joanne Nicholson, PhD, Professor
Institute for Behavioral Health
The Heller School for Social Policy and Management
Brandeis University, Waltham, Massachusetts

Kelly English, PhD, LICSW
Director of the Children's Behavioral Health Knowledge Center
Massachusetts Department of Mental Health
Boston, Massachusetts

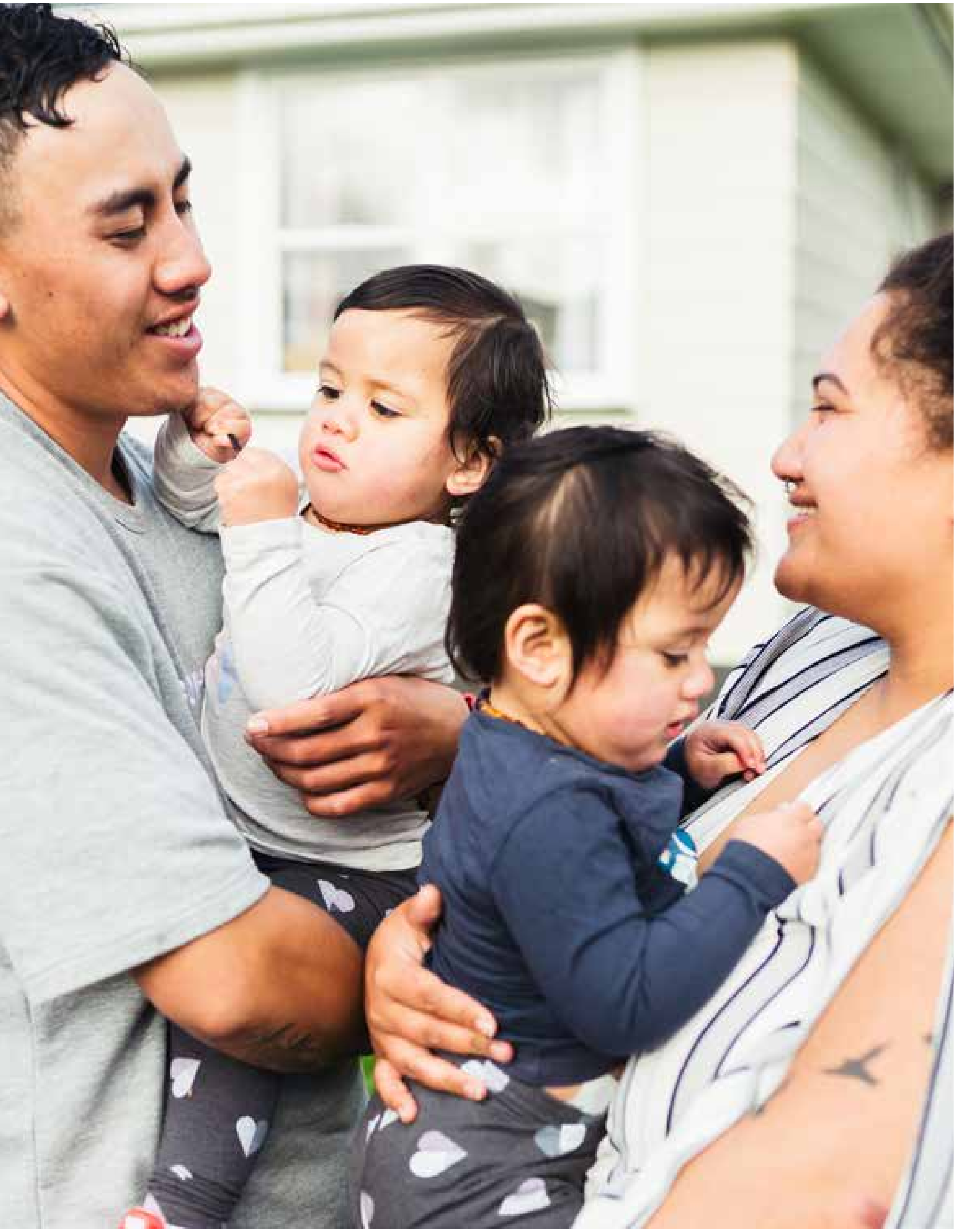

ParentingWell
PRACTICE PROFILE
EXECUTIVE SUMMARY

ParentingWell® is an approach to routine practice that makes talking about parenting, children, and family experiences a natural part of the conversation and of an adult's recovery process. Appropriate, effective treatment for an adult's specific behavioral health condition is paramount.

However, recovery does not occur in isolation. Family can provide motivation for change. Family members can encourage and support individuals to seek help. Parenting provides opportunities to be part of a community—in the neighborhood or through participation at children's school events, for example. ParentingWell promotes a parent's ability to live interdependently and to pursue recovery in the context of family life.

The ParentingWell practice approach promotes opportunities to conduct family-focused conversations, to generate a family-informed service plan, or simply to provide services to an adult living with a mental health condition or addiction, taking parenting and family circumstances into account. Just as you might routinely ask about housing, school or employment, or friends and social supports, it is important to ask:

1. Whether a person is or wants to be a parent,
2. How parenting and family experiences are going, and
3. How parents would like things to be.

ParentingWell is a versatile, responsive approach to a parent's situation and needs across the lifespan—meeting the parent "where they're at," when they're ready. You can implement conversations focusing on parenting and family life in an initial assessment or in the context of an ongoing relationship when the timing seems right and the parent is willing and able.

The initial objective of ParentingWell is achieved when the practitioner and parent work together to identify a parenting- or family-related goal and develop an action plan with steps for achieving this goal. Together, you can monitor progress and adjust the plan as necessary to insure success.

The goal may become part of the larger plan for services that the parent receives. Our hope is that parenting and family life become an ongoing part of the conversation over time. The key underlying principles of ParentingWell reflect a set of values shared by practitioners across service sectors: **family-focused, culturally-sensitive, strengths-based, and trauma-informed**.

FAMILY-FOCUSED:

The practitioner recognizes and respects the adult's unique experiences and expectations for parenting and family roles and responsibilities.

CULTURALLY-SENSITIVE:

The practitioner works actively to explore and understand the nuances and implications of a parent's cultural context and identity.

STRENGTHS-BASED:

The practitioner encourages and assists parents in identifying strengths to inspire hope for change and in building on strengths to set and achieve goals.

TRAUMA-INFORMED:

The practitioner is open to the likelihood that parents have experienced stressful life events and circumstances and helps them understand the relationship between past experiences, current functioning, and present family life.

The Core Elements and activities of the ParentingWell practice approach provide the framework for your conversations with parents. The Core Elements include: **Engage, Explore, Plan, and Access and Advocate.**

The purpose of the ParentingWell practice approach is to acknowledge that parenting is an important life role and to incorporate talking about goals for parenting and family life as part of the relationship routine. At best, ParentingWell becomes a part of the ongoing relationship and service delivery process.

Helping a parent succeed in this important life domain contributes to positive recovery outcomes.

ENGAGE

The first step is asking about a person's family situation and whether the person is, hopes to be, or has ever been a parent. Respectful, non-judgmental questions about parenting and family life promote normalization and community inclusion.

EXPLORE

Exploration involves asking how things are going currently regarding parenting, family life, and experiences with children and asking about past experiences to the extent they contribute to the person's current functioning as a parent, participation in treatment, and progress in recovery.

PLAN

Addressing adults' concerns as parents by helping them envision possibilities, assess options, and set and achieve goals is an essential focus of partnering with parents. Goals related to parenting and family life are woven into the parent's personal plan for change or into a formal treatment or service plan to support progress in the recovery journey.

ACCESS & ADVOCATE

Learning about whom or what the parent relies on for emotional, financial, spiritual, or concrete assistance helps to identify solid supports that can help parents to achieve their goals and cope with daily life and to address gaps where advocacy may help.

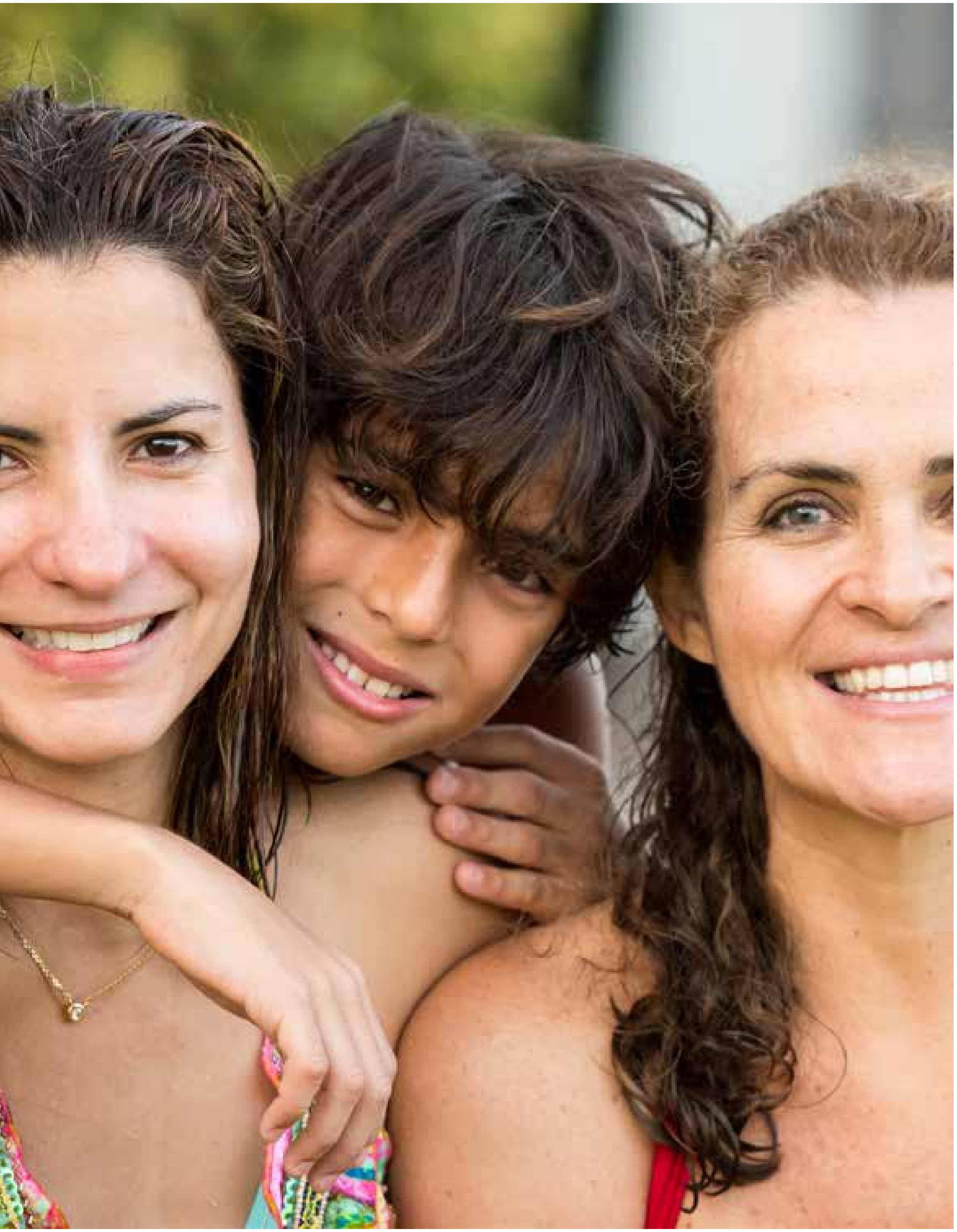

INTRODUCTION TO THE ParentingWell PRACTICE PROFILE

ParentingWell® is an approach to routine practice that makes talking about parenting, children, and family experiences a natural part of the conversation and of an adult's recovery process. While many practitioners may be inclined to consider and talk about the impact of a parent's mental health or addiction on his or her children, many are not trained to think about or to talk about the impact of parenting experiences and family life on an adult's behavioral health and recovery.

Consequently, practitioners may miss opportunities to provide support and guidance and to work together with parents to achieve their goals whether their goals are parenting related or not. Even if goals are not targeted to parenting and family needs or plans, parenting and family experiences can promote or undermine success in achieving goals in other domains, such as employment, education, and training or even getting to treatment appointments.

Appropriate, effective treatment for the adult's specific behavioral health condition is paramount. However, recovery does not occur in isolation. Family can provide motivation for change. Parents may want to do or be better for their children. Family members can encourage and support individuals in seeking help.

Parenting provides opportunities to be part of a community—in the neighborhood or through participation at children's school events, for example. Talking about family life is an opportunity, which often happens quite naturally, to take advantage of shared lived experiences in ways that can be helpful to everyone involved in the conversation.

Alternatively, family members and family relationships may throw up roadblocks to treatment, making scheduling of appointments difficult or even impossible, given the demands of family life. Significant family members or partners may not believe in treatment or may downplay a person's needs for support. Opening the door to talking about these issues may provide opportunities for problem-solving or troubleshooting to avoid barriers to effective treatment.

If you do not ask about parenting, you may overlook altogether what is important in a person's life. Research suggests that not considering parenting and family life may slow down an individual's progress in treatment or recovery and may, in turn, affect children's development and wellbeing. You may miss opportunities to promote engagement, to inspire motivation, to support strengths, and to build toward success in this valued life domain. Inadvertently, you may undermine hope and optimism. "Failing" as a parent may undermine an adult's optimal functioning and recovery altogether. Success as a parent may have important benefits in other significant life domains as well.

ParentingWell is not meant to be family therapy or necessarily to involve children as clients. It is not a standalone service but rather an approach embedded in other service, intervention, or support modalities, such as case management, individual therapy, peer supports, psychiatric rehabilitation, supported housing, or employment. The ParentingWell practice approach promotes opportunities to conduct family-focused conversations, to generate a family-informed service plan, or simply to provide services to an adult living with a mental health condition or addiction, taking parenting and family circumstances into account.

Just as you might routinely ask about housing, school or employment, or friends and social supports, it is important to ask whether a person is or wants to be a parent, how parenting and family experiences are going, and how they would like things to be.

You don't necessarily need to learn new skills. You can draw from and build on the skills you already have, keeping parenting and families in mind.

By attending to parenting and family experiences, the expectation is that your relationship with the person who is a parent will be enhanced. The parent will feel more hope and optimism. The person's capacity to attend to family issues will be improved and parenting stress reduced.

Over the longer-term, the parent will take further steps forward in their recovery journey—feeling better and doing better. These changes will, in turn, have positive impact on family relationships and resources and will contribute to improved outcomes for all family members.

ParentingWell may open the door to longer-term conversations with the adult, in the context of peer support, service delivery and treatment, that ultimately prevent the multi-generational transmission of behavioral health challenges.

The Goals of ParentingWell

In many ways, the goals of the ParentingWell practice approach are similar to those of all good practice, that is, individually-tailored, pragmatic, respectful of context and lived experience, and recovery-oriented. These include:

- To promote the development of a **trusting relationship** through open communication and mutual understanding.
- To lay the groundwork for wide-ranging **discussions about parenting and family life**, acknowledging the multi-directional nature of parent-child relationships and the many factors that contribute to recovery.
- To explore family experiences and circumstances, acknowledging **strengths as well as vulnerabilities**, and instilling hope that change is possible.
- To assist parents in specifying **relevant, realistic goals** with concrete steps and feedback loops, recognizing that parenting and family experiences may be a source of motivation and reward as well as a source of challenge and stress.
- To assess progress with parents, to deal with issues that may arise, to adjust goals accordingly, and to **acknowledge successes**.
- To encourage parents in developing and accessing **supports and resources**—both within the family as well as in the community.
- To support parents in **balancing family responsibilities with self-care and recovery**.

The ParentingWell practice approach focuses on three basic questions:

1. What are your parenting and family circumstances?
2. How are things going? AND
3. How would you like them to be?

The approach is versatile. You can implement conversations focusing on parenting and family life in an initial assessment or in the context of an on-going relationship when the timing seems right and the parent is willing and able.

The initial objective of ParentingWell is achieved when the practitioner and parent work together to identify a parenting- or family-related goal and develop an action plan with steps for achieving this goal. This goal may be set on a personal level or may become part of a more formal plan for services and supports the parent receives.

It is hoped that parenting and family life become an ongoing part of the conversation over time. In the best-case scenario, the practitioner and parent work together to monitor progress towards the goal, to adjust the goal or action plan when necessary, and to celebrate success when progress is achieved. Helping a parent succeed in this important life domain contributes to optimal functioning and positive recovery outcomes.

FAMILY-FOCUSED:

The practitioner recognizes and respects the adult's unique experiences and expectations for parenting and family roles and responsibilities.

ParentingWell Practice Principles

ParentingWell is a versatile, responsive approach to a parent's situation and needs across the lifespan—meeting the parent "where they're at," when they're ready. The key underlying principles reflect a set of values increasingly shared by practitioners across service sectors. These principles are woven through the Core Elements or activities of a ParentingWell approach with specific examples relevant to working together with parents provided in this Practice Profile.

CULTURALLY-SENSITIVE:

The practitioner works actively to explore and understand the nuances and implications of a parent's cultural context and identity.

STRENGTHS-BASED:

The practitioner encourages and assists parents in identifying strengths to inspire hope for change and in building on strengths to set and achieve goals.

TRAUMA-INFORMED:

The practitioner is open to the likelihood that parents have experienced stressful life events and circumstances and helps them understand the relationship between past experiences, current functioning, and present family life.

ParentingWell Core Elements

The Core Elements or activities of the ParentingWell practice approach provide the framework for your conversations with people who are already parents or those who want to become parents. These are: Engage, Explore, Plan, and Access and Advocate. The Core Elements may inform your approach at any point in your discussions with parents, particularly as you address the questions of whether or not the person is a parent, how things are going, and how they'd like things to be. They are not meant to be sequential. That is, you may find yourself working back and forth between Core Elements or addressing pieces of several Core Elements at one time.

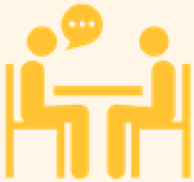

ENGAGE

Using the ParentingWell practice approach, the first step in establishing a positive, family-focused relationship with an individual is asking about their family situation and whether the person is, hopes to be, or has ever been a parent. Respectful, non-judgmental questions about parenting and family life along with other significant domains (e.g., work, education, and training) promote normalization, community inclusion, and the person's engagement with the practitioner, with the community, and with others who may offer support.

The process of relationship-building with a parent begins even before the person steps through the door. For example, respect is conveyed by the ways in which appointments are made and kept and in which phone calls are received and returned.

The purposeful sharing of your own experiences with a parent may help to build trust and confidence in the relationship. The initial engagement process lays the foundation for conveying and exchanging information, for making plans to address identified challenges and goals, and for promoting recovery and resilience.

Engagement is a process that continues throughout any relationship. Talking with parents about important life domains and attending to engagement over time will enhance your alliance with them.

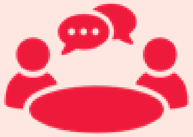

EXPLORE

The heart of the ParentingWell practice approach lies in the open flow of information and knowledge between practitioner and parent (or parent-to-be) to raise awareness about and to develop a shared understanding of the parent's situation and concerns, strengths and vulnerabilities, and priorities and motivation.

Exploration involves asking how things are going currently regarding parenting, family life, and experiences with children and asking about past experiences to the extent that they contribute to the person's current functioning as a parent.

As you and the parent get to know each other better, you will have opportunity to explore family experiences further, particularly as they relate to the parent's participation in treatment and progress in recovery.

Respectful curiosity contributes to relationship building; to the identification of strengths and vulnerabilities, supports and resources; and to addressing a parent's needs effectively.

PLAN

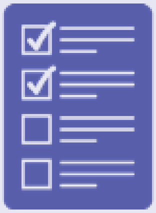

An essential focus of the ParentingWell practice approach is to address parents' needs by helping them envision possibilities, assess options, and set and achieve their desired goals. Goals related to parenting and family life may be woven into a personal plan to make desired changes or may become part of a more formal treatment or service plan to support progress in the recovery journey.

To create plans, help parents explore their motivation for change; picture desired outcomes; set goals; specify realistic, reasonable steps; identify resources and supports necessary to make progress; and determine appropriate time frames with feedback loops to adjust plans as necessary.

You can work in partnership with parents to explore options, to develop alternatives, and to think about the consequences of choosing one path over another. Parents may need information about existing but unknown possibilities, for example, community resources or entitlements. The key to a good plan is creating incremental opportunities for success—baby steps—that lead to the desired outcome.

ACCESS & ADVOCATE

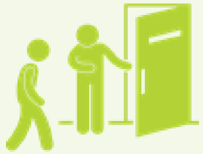

The ParentingWell approach to accessing essential supports and advocating for additional resources to meet parents' and families' needs begins with describing the current situation. Learning about supports and resources parents depend on for emotional, financial, spiritual, or concrete assistance (e.g., who might give them a ride to an appointment or babysit for a few hours) provides the foundation on which to build in the ParentingWell practice approach.

Advocacy may be required to fill in gaps. Parents benefit from the support of other parents often in the context of informal relationships, in neighborhoods, or at the playground. Community organizations, family, and friends offer parents opportunities to join in volunteering, play, learning, worship, and social activities.

Positive experiences contribute to resiliency and to a sense of mutuality and belonging. You can help parents learn skills that allow them to function better in relationships and in diverse situations through role modeling, rehearsal, and practice. As parents develop and navigate social networks and interpersonal situations better, they become more effective advocates for themselves and their children.

HOW TO USE THE PARENTINGWELL PRACTICE PROFILE

The ParentingWell principles and Core Elements are the essential ingredients of this practice approach. The following descriptions of each Core Element provide the basic definitions. Practice principles are translated into applications relevant to the Core Element.

Activities are suggested within each Core Element as a guideline for practitioners. These activities are not necessarily meant to occur in a particular order but are included in the description of each Core Element in the sequence in which they might be relevant. However, activities from different Core Elements may occur simultaneously, and you may work back and forth among all four Core Elements over time.

As you integrate these activities, a more complete picture of the person as a parent and their priorities for family life will emerge. You will be able to work with parents to help them weave their goals for parenting and family life into their vision for change and plans for the future.

FOLLOW-UP CONVERSATIONS

It is always possible to continue the ParentingWell conversation. In fact, this is the hope—that parenting and family life will be an ongoing topic of conversation in the helping relationship. On the other hand, a parent may choose to stop the discussion and take it up again in the future.

The purpose of the ParentingWell practice approach is to acknowledge that parenting is an important life role and to incorporate talking about goals and desires for parenting and family life as part of the relationship routine. At best, ParentingWell becomes a part of the ongoing relationship and service delivery process. Helping a parent succeed in this important life domain contributes to positive recovery outcomes.

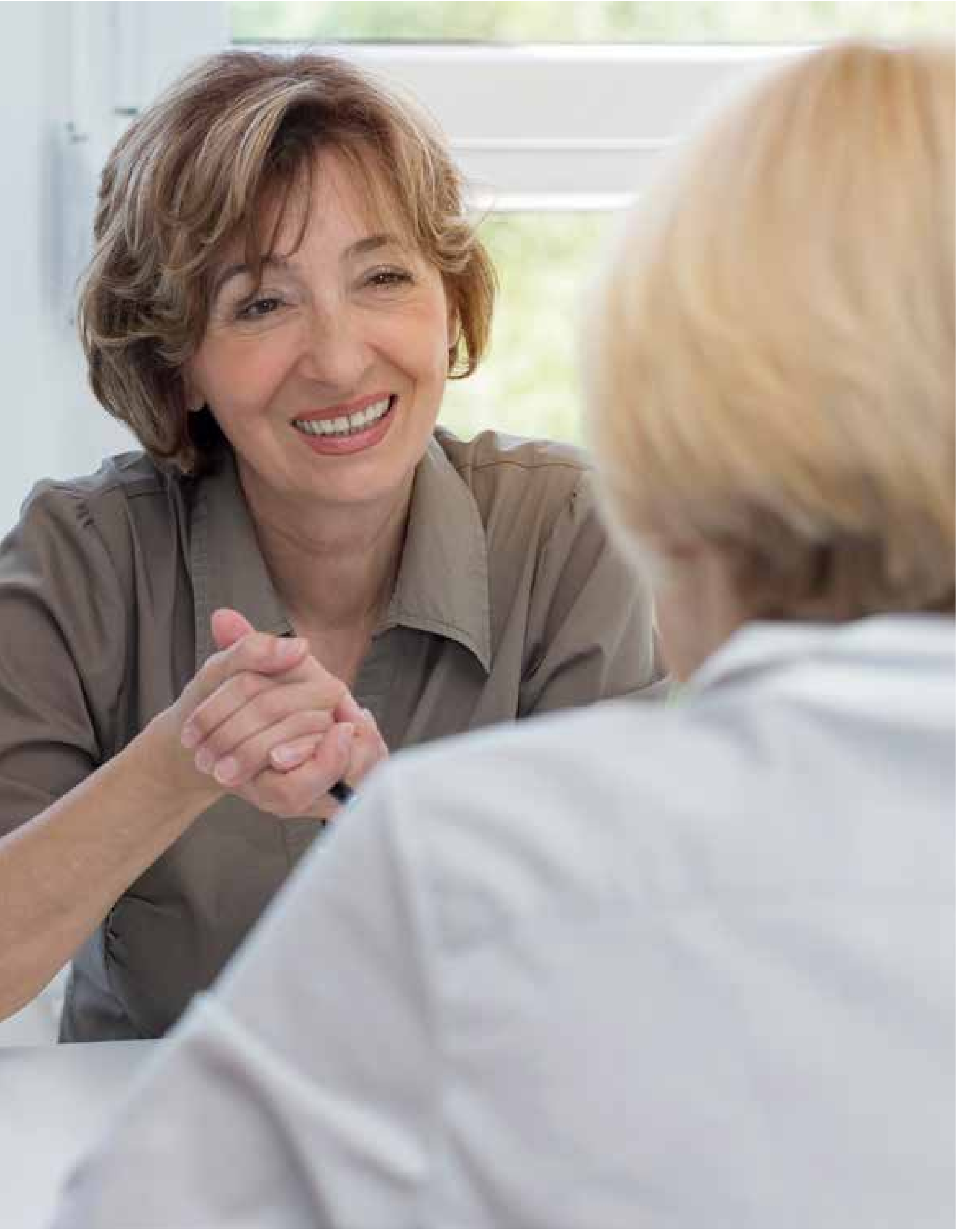

THE CORE ELEMENT OF
ENGAGE

Using the ParentingWell® practice approach, the first step in establishing a positive, family-focused relationship with an individual is asking about their family situation and whether the person is, hopes to be, or has ever been a parent. Respectful, non-judgmental questions about parenting and family life along with other significant domains (e.g., work, education, and training) promote normalization, community inclusion, and the person's engagement with the practitioner, with the community, and with others who may offer support.

The process of relationship-building with a parent begins even before the person steps through the door. For example, respect is conveyed by the ways in which appointments are made and kept and in which phone calls are received and returned.

The purposeful sharing of your own experiences with a parent may help to build trust and confidence in the relationship.

The initial engagement process lays the foundation for conveying and exchanging information, for making plans to address identified challenges and goals, and for promoting recovery and resilience.

Engagement is a process that continues throughout any relationship. Talking with parents about important life domains and attending to engagement over time will enhance your alliance with them.

TRANSLATING PRACTICE PRINCIPLES

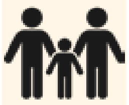

Family-focused

- Respects the individual's priorities and goals, including those related to parenting and family. The decision not to parent or relinquish custody may be the individual's choice, which must be respected. This path may involve feelings of success in making a well-considered personal choice, and/or feelings of loss and lost opportunity, which may be perceived because of a behavioral health condition.
- Acknowledges that the person may have previously unexpressed questions, concerns, and aspirations for present and future functioning as a parent and goals for family life.
- Recognizes that parenting is an important part of everyone's life—whether it is about having been parented or about being a parent.
- Stays alert to ways in which their own family experiences contribute to how they think about and respond to individuals as they describe parenting and family life.

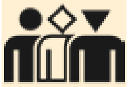

Culturally-sensitive

- Understands that cultural context and identity inform expectations for and experiences of family life as well as opportunities for family and community inclusion or isolation.
- Reflects on the ways in which culture and identity shape how individuals assess and frame their behavioral health and treatment experiences; how they think and feel about themselves; and how they take care of their children.
- Realizes that discussing cultural considerations with each person highlights differences and similarities with the practitioner's own culture that may either enhance or interfere with the relationship.

CORE ACTIVITIES

- Contacts the person/parent prior to the face-to face meeting to (re) introduce themselves and clarify their role, to remind the person/parent about the appointment, to ask them how they want to be addressed (e.g., first name, Mr. or Ms., pronoun preference, etc.), and to offer to answer any questions the person/parent might have.
- During the first meeting, welcomes the person and asks initial questions about parenting and family status (e.g., Are you a parent? Do you have children?).
- Explains the rationale for asking questions about family members and relationships, especially as they relate to children, and for understanding that the questions may raise positive feelings along with negative feelings or challenges.
- Identifies family and household members , including children of any age and legal status, if known, along with a co-parent, if there is one.
- Determines the person's pregnancy status or future hopes and plans for children and family.
- Ascertains where children are living and who is caring for them.
- Obtains information regarding custody and visitation arrangements, if relevant, and the extent of the parent's contact with children.
- Asks how the person experiences conversations about parenting and family life. Addresses and respects feelings, considering parent's concerns about content and pacing of conversations.
- Acknowledges and addresses any reluctance the person may have to talk about parenting and family issues at this time.

TRANSLATING PRACTICE PRINCIPLES

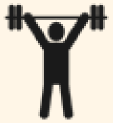

Strengths-based

- Understands that a welcoming, responsive, respectful approach conveys hope, promotes empathy and acceptance, and builds trust.
- Strives to provide non-judgmental responses to the person’s expression of challenges in caring for children and balancing recovery with family life.
- Questions their own assumptions about how well people living with behavioral health conditions can parent and if it is possible to believe they have strengths and can realistically hope for change.

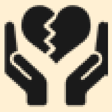

Trauma-informed

- Recognizes that previous relationships may have been traumatic. Parents with behavioral health conditions may have had negative experiences with practitioners (and systems) in the past and may have difficulty trusting a new practitioner.
- Considers that the person may be worried about how the practitioner will respond in talking about parenting issues (e.g., if the person has had other practitioners who expressed negative attitudes about their capacity to parent).
- Realizes that trust may only be built over time but that asking the initial questions opens the door to future, important conversations and relationship-building.

CORE ACTIVITIES

(Continued from previous page)

- Provides parents with an understanding of the ways in which their health and wellbeing may affect their parenting and children.
- Suggests ways in which their parenting and family experiences affect their own wellbeing and recovery.
- Provides information and/or access to resources about children’s developmental ages and stages when opportunities arise or if parents ask questions or express concerns.
- Provides transparency regarding risks and vulnerabilities, mandated reporting, and the ways in which these issues, should they arise, will be handled in collaboration with the parent to achieve the shared goal of family wellbeing and safety.
- Encourages the person/parent to talk about parenting, children, and family relationships in future sessions.

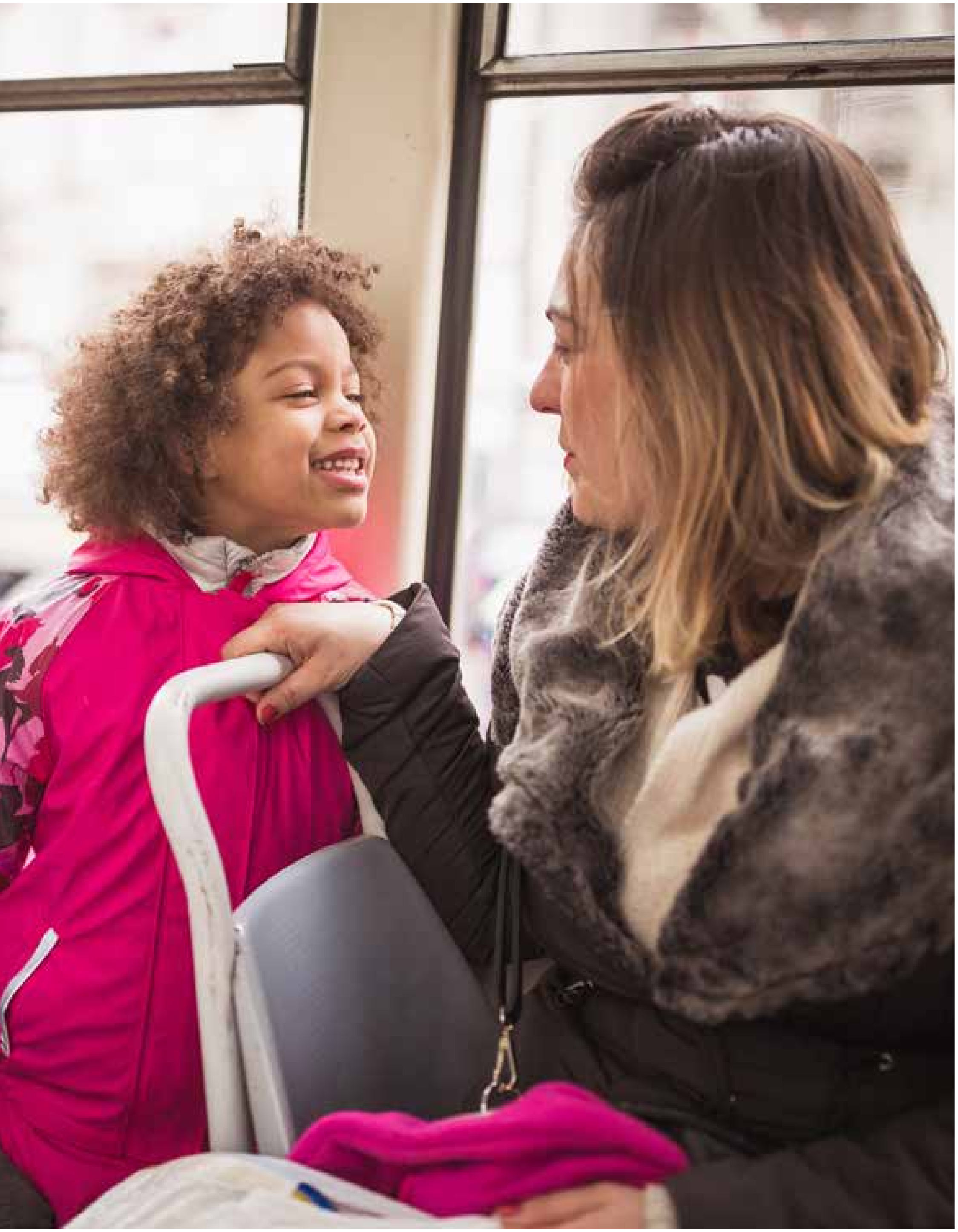

THE CORE ELEMENT OF

EXPLORE

The heart of the ParentingWell® practice approach lies in the open flow of information and knowledge between practitioner and parent (or parent-to-be) to raise awareness about and to develop a shared understanding of the parent's situation and concerns, strengths and vulnerabilities, and priorities and motivation.

Exploration involves asking how things are going currently regarding parenting, family life and experiences with children, and asking about past experiences to the extent they contribute to the person's current functioning as a parent.

As you and the parent get to know each other better, you will have opportunity to explore family experiences further, particularly as they relate to the parent's engagement in the relationship, participation in treatment and progress in recovery.

Respectful curiosity contributes to relationship building; to the identification of strengths and vulnerabilities, supports and resources; and to addressing a parent's needs effectively.

TRANSLATING PRACTICE PRINCIPLES

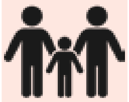

Family-focused

- Views adults not only in terms of behavioral health symptoms, diagnosis, and illness management, but also in terms of roles, responsibilities, and priorities as parents and family members.
- Acknowledges that parents are the experts on their children and family life. Respects the information and questions they have.
- Recognizes that parents benefit from understanding children's common worries about their mental illness or addiction and from knowing how to have conversations with children about their behavioral health, treatment, and recovery.
- Seeks support in understanding their own attitudes and emotional responses when talking about parenting, children, and families.

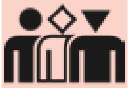

Culturally-sensitive

- Identifies experiences, strengths, and resources related to cultural context and identity (e.g., LGBTQ community supports, extended family, faith community).
- Understands that cultural context and identity inform consideration of a person's social network and social support.
- Recognizes that discussing culture and identity with a parent highlights differences and similarities with the practitioner's own background and beliefs that may either enhance or interfere with the relationship.

CORE ACTIVITIES

- Begins the conversation with "How are things going?" in general, and then more specifically, how things are going with children and family life.
- Discusses daily routines, household chores, and taking care of the children. Suggests describing a typical day to provide the parent support and structure for talking about these issues, if this will help.
- Anticipates a parent's hesitancy, responds sensitively, and supports the parent in sharing their experiences in a positive way.
- Listens carefully and is genuinely curious as parents talk about their experiences, strengths, and vulnerabilities and those of their children.
- Makes brief notes about children's situations, development, and functioning in relevant life domains (e.g., school, with peers, at home, etc.).
- Guides the parent in understanding the bi-directional nature of parent's and children's wellbeing.
- Provides information in response to adult's questions about behavioral health and parenting.
- Asks about a parent's understanding, in situations of custody loss or limited contact with children, of why/how this happened and how the parent is coping (e.g., possible feelings of loss, grief, pain, relief). How do children understand the separation? How are they coping?

TRANSLATING PRACTICE PRINCIPLES

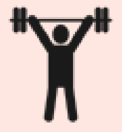

Strengths-based

- Recognizes that strengths inspire hope and help a person get through the day and that identifying and building on strengths demonstrates respect for the parent and leads to success.
- Understands that parents, especially those who are quite depressed or see themselves as “failures,” may require assistance in identifying strengths and resources in themselves and in their children.

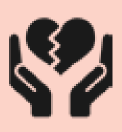

Trauma-informed

- Considers and respects the parent’s efforts to control the content and pace of questions and answers.
- Anticipates that parents with behavioral health conditions are vulnerable to stigma and may have experienced the negative attitudes of others.
- Reflects on their own skills and knowledge and the emotional content of the work to recognize issues that may trigger the practitioner’s own traumatic experiences.

CORE ACTIVITIES

(Continued from previous page)

- In situations of loss of custody or contact with children, asks about parent’s understanding of why/how this happened and how the adult/parent is coping (e.g., possible feelings of loss, grief, pain). How do children understand the separation? How are they coping?
- Supports the parent in identifying strengths and resources, particularly as they relate to parenting/relationships with children and family life, social support, and self-care.
- Asks about any history of trauma and consequent impact (e.g., significant losses, deaths of loved ones, homelessness, abuse or violence, etc.).
- Returns to the topic of traumatic experiences in general when the parent is ready and willing to discuss.
- Asks about cultural norms and family beliefs regarding behavioral health concerns and treatment.
- Asks about beliefs regarding parenting, expectations for children, and child behavior management.
- Asks parent if they have talked with their children about their behavioral health and treatment and what their children witness, perceive, and understand. How do children interpret the symptoms and behaviors of their parent?
- Discusses children’s common worries and fears about parent’s condition (e.g., Did I cause it? Will I catch it? Can I fix it?).
- Discusses the perspectives of older/adult children (e.g., anger, disappointment, resilience, empathy, success).

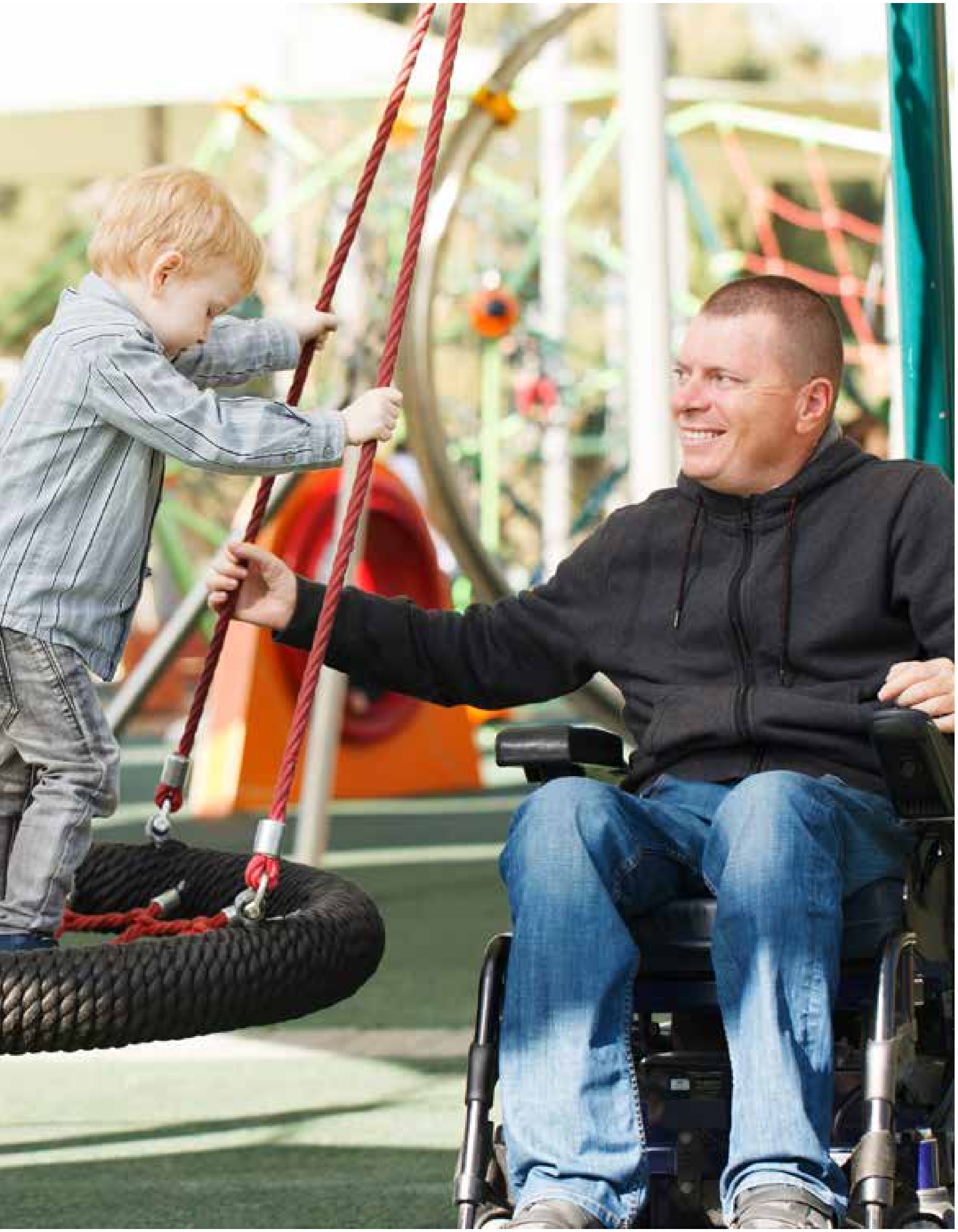

THE CORE ELEMENT OF PLAN

An essential focus of the ParentingWell® practice approach is to address parents' needs by helping them envision possibilities, assess options, and set and achieve their desired goals. Goals related to parenting and family life may be woven into a personal plan to make desired changes or may become part of a more formal treatment or service plan to support progress in the recovery journey.

To create plans, help parents explore their motivation for change; picture desired outcomes; set goals; specify realistic, reasonable steps; identify resources and supports necessary to make progress; and determine appropriate time frames with feedback loops to adjust plans as necessary.

You can work in partnership with parents to explore options, to develop alternatives, and to think about the consequences of choosing one path over another. Parents may need information about existing but unknown possibilities, for example, community resources or entitlements.

The key to a good plan is creating incremental opportunities for success—baby steps—that lead to the desired outcome.

TRANSLATING PRACTICE PRINCIPLES

Family-focused

- Understands that parents should be asked about their priorities, particularly as they relate to family life, parenting, wellbeing, and recovery.
- Recognizes that parents are free to decide how they will act and what steps they will take to address their priorities, knowing that they may have to comply with the expectations of child welfare services or family court.
- Acknowledges that balancing parenting responsibilities with taking care of one's self can be challenging for most parents, even if the parent doesn't have a behavioral health condition.
- From the parent's perspective, understands that making the choice to prioritize the parent's own needs may seem contrary to taking care of their children, may contribute to feelings of guilt or shame, but may be essential to improving the parent's coping skills and resilience.

Culturally-sensitive

- Recognizes that parents' priorities may, in part, be influenced by the expectations of others in the family or community.
- Understands that parents may set priorities that are not consistent with the attitudes and beliefs of practitioners.

CORE ACTIVITIES

- Helps parents identify what they want to change and picture the outcomes.
- Assists parents in setting priorities.
- Helps parents identify options and consider the benefits or costs of choosing one option over another.
- Works together with parents to set goals that are "SMART"—specific, measurable, actionable, relevant, and time-bound.
- Discusses the value of following up on implementing the plan, evaluating, and revising or setting a new goal to continue moving forward.
- Assists with a problem-solving approach if parents cannot "put the pieces in place" to take steps forward.
- Debriefs with parents when goals are not achieved to discuss options, to make a new plan, and to use lessons learned to move forward.
- Helps parents anticipate crises and address vulnerabilities through preventive action (e.g., developing a plan for coping with an anticipated challenge) or activating resources (e.g., identifying someone who could help the parent).

TRANSLATING PRACTICE PRINCIPLES

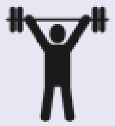

Strengths-based

- Understands that progress is measured in baby steps.
- Does not judge but offers guidance and feedback to keep parents' priorities, goals, and solutions realistic, reasonable, and safe.
- Role models good self-care for parents by taking care of themselves.

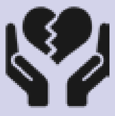

Trauma-informed

- Understands that parents may have options but may not have experience making good choices.
- Recognizes the importance of parents building resilience and developing coping skills for themselves to serve as role models for their children.
- Understands that trauma survivors may have to practice healthy ways of coping to replace unhealthy ways of coping from the past.

CORE ACTIVITIES

(Continued from previous page)

- Provides concrete suggestions for time management and other organizational skills essential to achieving goals successfully.
- Assists parents to develop strategies for keeping in touch with children when they are apart, possibly due to a hospitalization, custody issues, or out-of-home placement or when adult children live independently.
- Helps parents make back-up plans for providing for children if and when symptoms emerge or if parents need to be hospitalized.
- Provides the rationale for taking care of oneself—to be able to take care of one's children better. Asks about current self-care strategies and routines.
- Checks-in regularly to help parents evaluate progress and adjust goals or action steps, if necessary.
- Supports parents in identifying ways to celebrate small steps and large accomplishments.

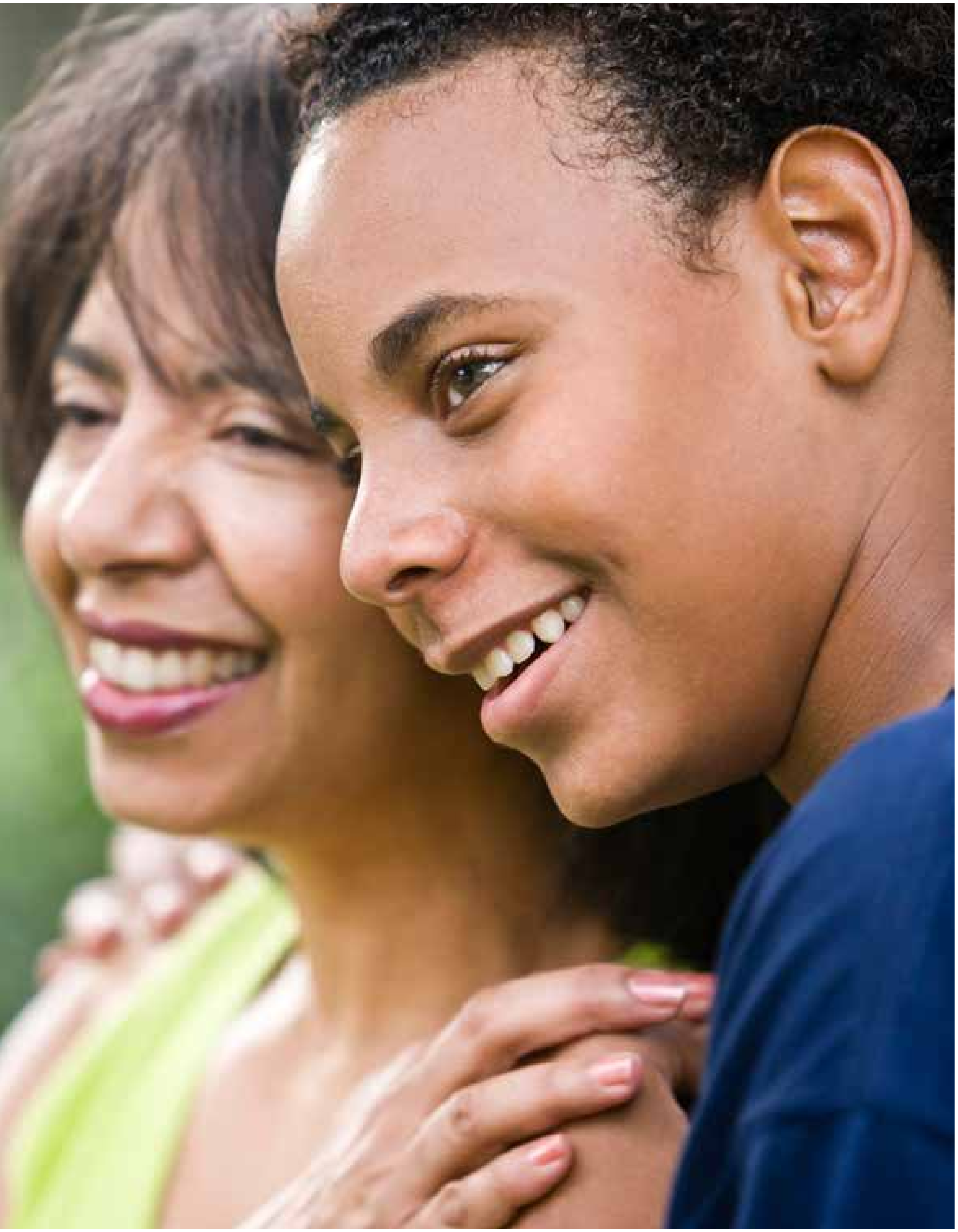

THE CORE ELEMENT OF

ACCESS & ADVOCATE

The ParentingWell® approach to accessing essential supports and advocating for additional resources to meet parents' and families' needs begins with describing the current situation. Learning about supports and resources parents depend on for emotional, financial, spiritual, or concrete assistance (e.g., who might give them a ride to an appointment or babysit for a few hours) provides the foundation on which to build in the ParentingWell practice approach.

Advocacy may be required to fill in gaps. Parents benefit from the support of other parents often in the context of informal relationships, in neighborhoods, or at the playground. Community organizations, family, and friends offer parents opportunities to join in volunteering, play, learning, worship, and social activities.

Positive experiences contribute to resiliency and to a sense of mutuality and belonging.

You can help parents learn skills that allow them to function better in relationships and in diverse situations through role modeling, rehearsal, and practice. As parents develop and navigate social networks and interpersonal situations better, they become more effective advocates for themselves and their children.

TRANSLATING PRACTICE PRINCIPLES

Family-focused

- Understands that parents benefit from the support of other parents often provided in the context of informal relationships, in families, in neighborhoods, or at the playground.
- Recognizes that social support both personal and professional enhances the likelihood of positive outcomes for adults who are parents and for their children.
- Does not assume that immediate relatives or extended family members are necessarily helpful as they may have attitudes about mental illness or addictions, parenting and family life that are unsupportive of the parent.

Culturally-sensitive

- Appreciates that an understanding of the parent's cultural and community context will help the practitioner, working together with the parent, to identify available interpersonal, informal, and professional resources.

CORE ACTIVITIES

- Begins the conversation about current supports and resources—emotional, financial, instrumental, professional, and personal—that benefit the parent, the child, and the family.
- Provides the rationale for the benefits of social support and positive interactions for both parents and children as they contribute to recovery and resilience.
- Suggests the benefits of and supports parents in helping others as well as asking for help as mutual give-and-take builds more supportive relationships.
- Explores any hesitancy on the part of parents to connect or communicate with others regarding parenting or children, especially to ask for help.
- Asks who helps most with child caregiving, if relevant. Who do parents ask for advice about parenting and relationships with children?
- Asks parents about strengths of partners or other significant family members, especially those who may be involved as caregivers.
- Asks how often and in what context parents interact with other adults (e.g., friends, neighbors, family members), especially other adults who are parents.
- Assists parents in identifying individuals or family members of their choosing, who have some understanding of their circumstances, who can or do serve as positive role models, or who provide positive assistance to the parent and family.
- Asks about resources available or used in the neighborhood and community.

TRANSLATING PRACTICE PRINCIPLES

Strengths-based

- Recognizes that connections and communication are essential as recovery takes place in the context of relationships and that parents may need help with skills to promote these.
- Accepts that all parents benefit from help, have questions, and seek advice. It is normal and normalizing to ask for help.
- Understands that parents may need encouragement to reach out to others.
- Appreciates that parents may require support to increase the frequency of positive interactions with children.

Trauma-informed

- Understands that parents may be afraid to ask for help due to the stigma associated with mental illness and substance use and the fear of being judged negatively.
- Recognizes that parents may need guidance to talk with children, family members, and professionals about important topics related to behavioral health issues and parenting, especially if these conversations have been unsatisfying or unsuccessful in the past.

CORE ACTIVITIES

(Continued from previous page)

- Asks about involvement with any other systems, services, or practitioners (e.g., child welfare, child's therapist, school system, other services).
- Supports parents in influencing people and environments that promote recovery for themselves and resiliency for their children, from the practical (e.g., negotiating with a landlord for safety latches on screens or cabinet doors) to the professional (e.g., advocating for special education services for children in the context of a school meeting).
- Encourages, role models, coaches, and offers parents opportunities to rehearse and practice interacting with others, particularly if the parent is not socially adept, prefers to be alone, is socially fearful, or feels inadequate or as if no one else understands their situation.
- Helps parents identify alternative or new social support resources, specifically with regard to parenting and family life.
- Researches available opportunities for social support (e.g., parent groups) with the parent.
- Assists parents in identifying professional sources of support, particularly as they may help with parenting issues (e.g., pediatrician, school teacher or counselor, parent support groups).
- Supports parents' developing skill, growing confidence in interpersonal situations to promote continued development, maintenance of social networks and supports, and access to essential resources.

Glossary

This brief glossary is provided to insure a shared understanding of the words used in the ParentingWell Practice Profile.

Practitioner
A person in any role working together with or providing services to an adult who is, has ever been, or hopes to be a parent. A practitioner can be a clinician, social worker, case manager, outreach worker, or peer specialist. A person may be a practitioner by training and expertise or by the lived experience of behavioral health conditions and recovery. You typically think of a practitioner as partnering with a parent, that is, working directly together to talk about experiences, to explore priorities and needs, to make plans, and to access resources and supports. **However, any person who comes into contact with parents has opportunities to engage them through a ParentingWell approach**. The administrative assistant who takes a telephone message can treat a parent with dignity and respect. The program manager who sees a parent in the agency hallway can convey a welcoming, non-judgmental attitude.

Parent
A person who has ever given birth to, fathered, adopted, fostered, step-parented (officially or informally), or served as a long-term caregiver to a child of any age. We take a broad perspective to this definition because parents, especially those with behavioral health conditions, can find themselves in diverse, dynamic situations in relationships with children of varying ages and developmental stages.

Family
A set of individuals (adults and children) who define themselves as a close-knit group because of biological, marital/partner relationships, friendships, or living circumstances. Family members may live together or be separated by distance or by lifestyle choices. Their relationships may be supportive and comforting or conflict-laden and challenging.

Behavioral Health
A mental illness, a substance use disorder or addiction, or a combination. We use behavioral health as an umbrella term to encompass all of these. While there are nuances and differences in parents' experiences depending on their assigned diagnoses, treatment regimes, and recovery journeys, their experiences with parenting, children, family life, and professional services may share many similarities.

Lived Experience
The experience of living with a mental health or substance use disorder or addiction. People's experiences change over time with life circumstances, participation in treatment and services, and varying commitment and capacity to pursue recovery.

As a practitioner, your own lived experience informs and contributes to the work you do. It is important that you take time to reflect on your experience of family life and to consider the ways you interact with parents with care and thought, purpose, and intent.
