# Supplementary material for: The ParentingWell Practice Approach: Adaptation of Let’s Talk About Children for Parents With Mental Illness in Adult Mental Health Services in the United States
Source: Front Psychiatry. 2022 Apr 7;13:801065. doi: 10.3389/fpsyt.2022.801065 (PMC9021592; doi:10.3389/fpsyt.2022.801065)
Supplement: Supplementary file 2 [file Data_Sheet_2.PDF]

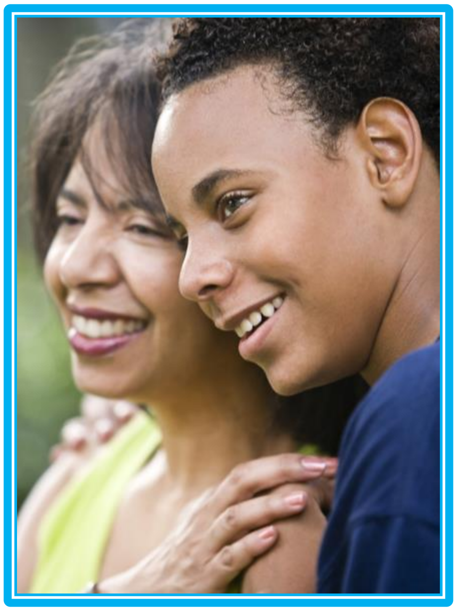
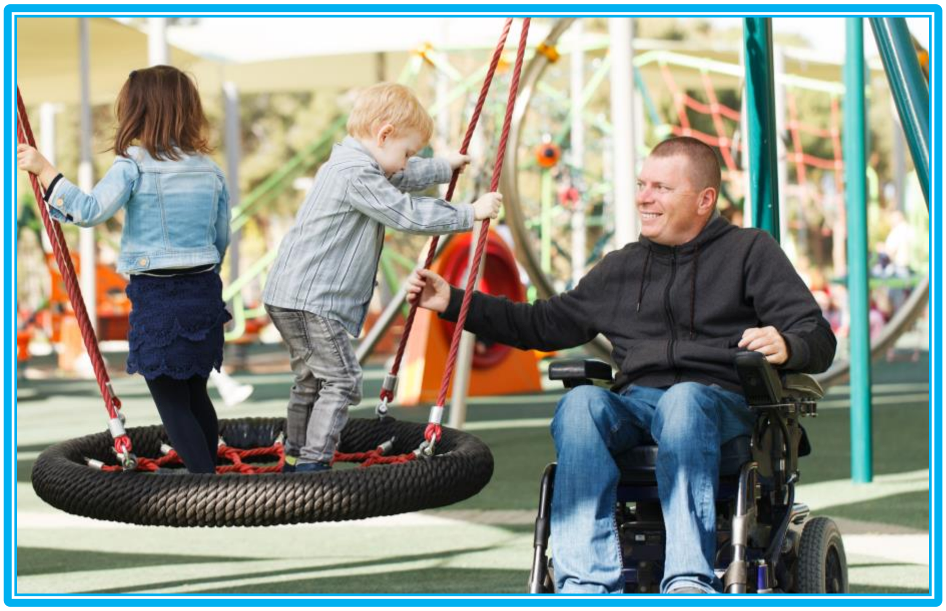
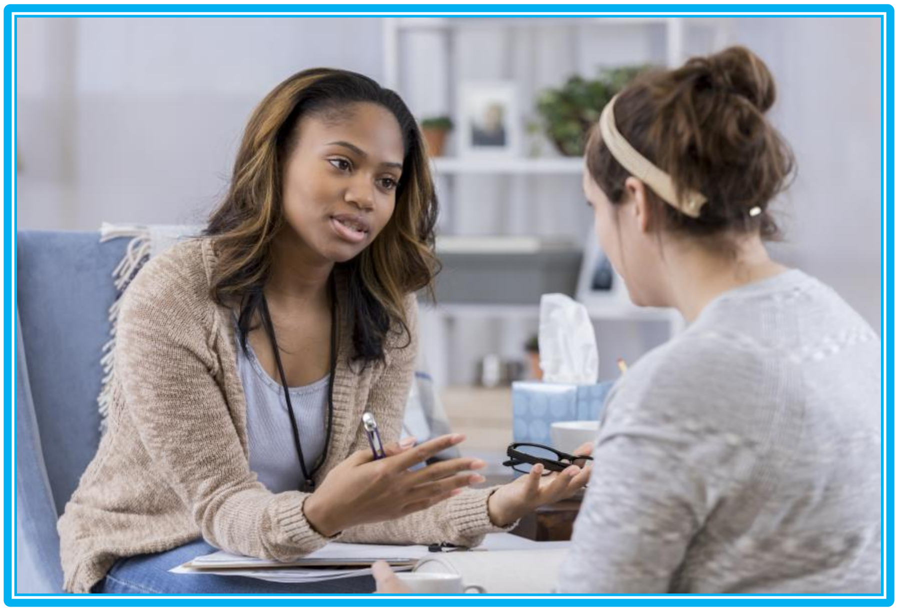
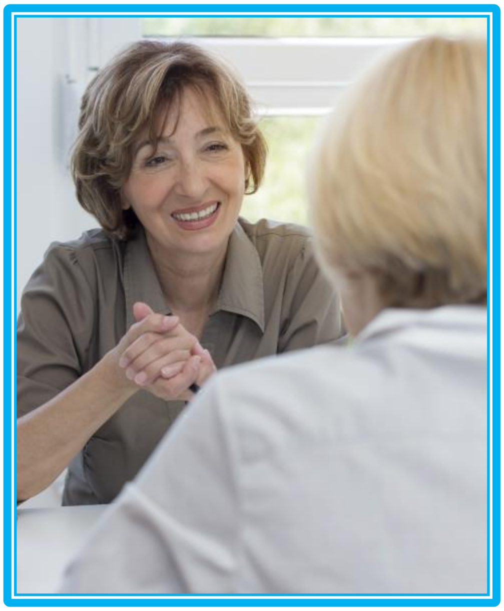

ParentingWell Workbook

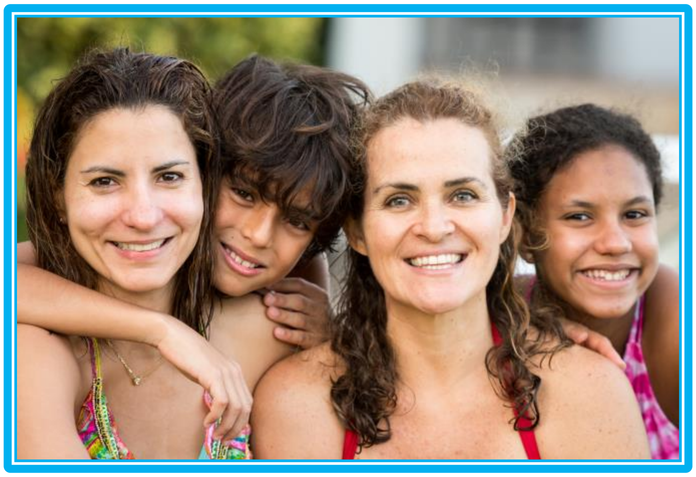
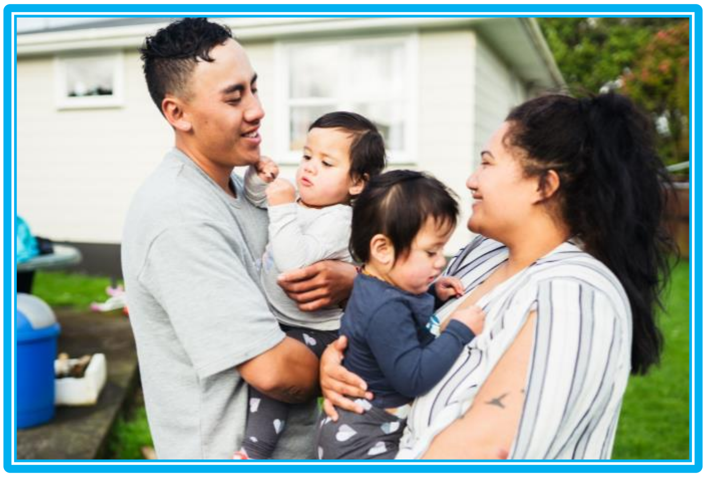

APPLYING SKILLS, BORROWING TOOLS, AND USING RESOURCES

Depending on your training and experience, you may not have to learn any new skills or tools to embrace a ParentingWell practice approach. If you are trained clinician or a certified peer specialist you have undoubtedly learned and practiced strategies for talking with and engaging “clients” or “consumers” or peers in addressing their needs. You can use these same skills as you tailor them to parents and their circumstances. If you are not familiar with any of these skills or tools, seek out situations to learn and practice them, with peers or your supervisor. Resources are provided throughout the ParentingWell practice profile, along with coaching materials to help you.

Providing Information

Parents may request or benefit from diverse types of information, from psycho-education regarding their behavioral health conditions, the multi-directionality of parent-child relationships, and the ways in which parenting responsibilities and experiences of family life contribute to the recovery process, to information about available community resources or instruction about how to access essential services. You may provide information on anticipated consequences of a particular choice or action on the parent’s part, or what another person’s perception of the choice or action might be.

EXAMPLE

“Your mental illness does not necessarily mean your child will have a mental illness. There are things you can do to help your child grow up well. We can talk about these.”

POSING QUESTIONS

How you ask a question is just as important as what you ask. You may sound curious or confrontative, depending on what is appropriate to the situation. Your questions may uncover a parent’s motivation for change or help identify barriers to achieving a goal.

 © 2013 ParentingWell. All rights reserved.

EXAMPLE

"Let's think about what will happen if you miss your treatment appointment. How important is your mental health in your relationship with your children? Are there ways in which neglecting your own care might affect them? What is keeping you from attending your appointment? What would help you get there?"

Reflecting

Helping a parent reflect on his or her experiences, thoughts or feelings can open up positive avenues to change. It can also help you understand what is really "going on" for the parent, to inform or confirm the assumptions you may be making about what the parent is saying. Parents who are prone to ruminating may benefit from help with setting limits on thinking too much about something, possibly by considering strategies for distracting or diverting the thought process with action.

EXAMPLE

"You'd like to have more positive phone conversations with your children. What happens in these calls that you would like to change? What makes you start crying? What would help you with that?"

Reframing

Reframing requires you to shift the parent's perspective, and quite possibly your own, from one point of view to another. This does not mean you avoid or ignore a situation if the parent is not doing well or if things are going poorly. Rather, reframing allows the parent see a situation differently, to move forward for example, rather than dwell in the past.

EXAMPLE

"You think you're a failure because you gave up your children for adoption. I suspect that required great strength on your part. You really prioritized their needs at the time—a sign of good mothering."

 © 2013 ParentingWell. All rights reserved.

Perspective-taking

Many people have a hard time taking the position of another person, particularly if their lived experience is much different. Perspective-taking requires an individual to "walk in another person's shoes." For a parent, the other person may be a child, a partner or grandparent, or a child welfare worker.

EXAMPLE

"I know there are many days when you are depressed and crying. What might your 8-year-old think about that? Many children think that if their parent is upset, it's their fault – and that they can fix things for you. Your child might have less difficulty going to school in the morning if you explained to her that you are feeling 'down' and that it's not something she can fix; that you have a therapist who is helping you take care of things and feel better."

Role Modeling and Rehearsing

People often learn best by watching someone else do something and then trying it themselves. For example, parents with behavioral health conditions may have great difficulty advocating with teachers or school counselors in an effort to get their children the educational services they need. You can role model appropriate advocacy skills, perhaps with a parent sitting next to you while you make a phone call to the school or participate together in a team meeting. You can rehearse or practice specific skills or a particular situation, for example, asking for help without getting angry or upset.

EXAMPLE

"Ok, let's try this out. First, you be the teacher and I'll be you, asking for help with your child's homework. Then, I'll be the teacher and you ask me for help. I know you feel the teacher blamed you in the past for not helping your son with his math. Let's see if we can come up with what to say and do, so you don't start the conversation feeling so angry or ashamed that you don't accomplish your goal."

 © 2013 ParentingWell. All rights reserved.

Recognizing Patterns, Changing Habits, and Building Skills

Everyone gets stuck in "vicious cycles" or patterns of thoughts, feelings and behavior they'd like to change. Parents may identify habits or triggers and responses they would like to modify. You can find any number of tips for changing habits on the Internet. They all involve similar strategies of observing and maybe even recording the cycle or pattern of thoughts, feelings and behavior; experimenting with alternative ways of responding; and then paying attention to the results. Parents may need help in developing both the skills for expressing themselves as well as the capacity to listen, to respond to the cares and concerns of others. Changing habits or building new skills to replace old ones that don't work so well requires practice, practice, practice.

EXAMPLE

"I always scream at my kids on the phone when they don't want to talk to me. I get mad when they call their foster mother "mom" – she is not their mother - and then I start yelling, and they hang up…" You may want to help the parent take a closer look at the patterns involved in this situation, brainstorm together about alternative ways of responding or handling the situation to achieve the parent's desired outcome, and then practice together. The parent can then try out the new skills in a "real-world" conversation and report back for feedback and guidance, and more practice.

Problem-solving and Goal-setting

Parents may need help envisioning desired changes, identifying the necessary resources (including motivation, time and energy) and potential barriers, possibilities and next steps through a problem-solving approach or a collaborative mapping of the vision for change and desired outcomes. The biggest challenge facing many parents living with behavioral health conditions is balancing self-care with taking care of others. This is counter-intuitive to many parents—don't children always come first if you are a "good" parent? Parents can be encouraged to consider ways to take care of themselves that allow them to take the steps towards achieving their goals for parenting

 © 2013 ParentingWell. All rights reserved.

and family life. Solutions are identified, integrating and building on strengths to achieve reasonable, realistic goals. The key to these tasks is to create opportunities for success, which may be defined differently by different people. Benchmarks should be set along the way to mark progress and to set a time to re-evaluate and make a new plan, if necessary. Remember, change happens in baby steps.

EXAMPLE

"My problem is that I get really stressed out fixing supper. The kids are supposed to be doing their homework and not watching tv, and I have to keep yelling at them and they won't settle down, and then I have to have a glass of wine or two to calm myself down and that becomes three or four glasses until dinner is ready. Sometimes I just give up on dinner and give them bowls of cereal…and I keep drinking. Then they have to put themselves to bed…" The practitioner can help the parent envision the way she would like things to go, identify barriers and supports, list realistic steps towards her envisioned goal, monitor progress and provide feedback.

 © 2013 ParentingWell. All rights reserved.

REFLECT ON YOUR OWN LIVED EXPERIENCE

Steps you can take

- **Consider your parents.** Even if you are not a parent yourself, you have parents. Take a moment to think about them and yourself in this role. On a scale from 1 to 10, say, how important is being a parent to you? How important were or are your parents in your life—whether your experience was positive or negative or, most likely, a combination of both? If you are a parent—where do you turn for help when you are feeling tired or overwhelmed? If you are not a parent, consider your own childhood. Were there others, besides your parents, who provided guidance, nurturance and support? All parents need partners, that is, others who may provide babysitting or day care; social, recreational or creative opportunities for children; or who help to insure children's emotional safety and spiritual development. Who were your parents' partners—in your family, in your community? Who are yours?
- **Think about your childhood.** It is easy to find fault with our own parents. This may even be an expectation, say, in conversations with friends, relatives or professional service providers. It may be easy to rattle off a list of offenses—ways in which your parents disappointed you. Many adults who become parents themselves are driven by the belief that they will "never do to my children what my parents did to me." It may be more challenging to identify ways in which your parents were nurturing, supportive and helpful to you. It is extremely important to identify your parents' positive traits, or characteristics and strengths to have a positive image of yourself. By acknowledging the positive things your parents brought to your family relationships, along with or even in spite of the negative, you will begin to form a more complete picture of yourself, yourself as a parent, and the parents with whom you work.

 © 2013 ParentingWell. All rights reserved.

- **Consider your own parenting style.** How would you describe yourself? Do you consider yourself permissive or strict? Are you more of a “friend” to your children or the “boss” of the family? It is easy to fall into describing ourselves at the positive or negative end of a continuum. Consider the entire continuum, and the times you may lean towards one end or another. Keep an open mind about another person’s approach to parenting and remember there are few completely right or wrong approaches or answers.
- **Reflect on your own experience of health and wellness.** You may or may not be a person with lived experience of a behavioral health condition. But everyone has their ups and downs, times of calm and contentment, and times of stress and distress. And everyone benefits from insight into knowing what helps them feel more relaxed, better or happier, and what can stress them out or when they feel bad.
    - Notice times when you feel depressed, anxious or distressed, and times when you feel happy, relaxed and confident, and make brief notes in a log for one week.
    - Select one example of a time when you felt great and a time when you felt not so great.
    - Can you identify the events, experiences or circumstances surrounding each of these examples—both feeling good and feeling bad?
    - For when you were feeling good—what specifically was it about this time that felt good? When you were feeling bad, what was at the root of this experience? What helped you feel better afterwards?
    - What can you learn from this reflection that you might apply in a future situation?
    - Try out this strategy a few times in the coming days. Was it helpful? How might you adjust the strategy to fit and feel better?
    - This exercise may help you identify some of your own risk and protective factors.

 © 2013 ParentingWell. All rights reserved.

ENGAGE:
Consider the parent's experience

It is important to consider a parent's experience of your practice setting and procedures—from the moment they or you make that first phone call to set up an appointment to when they or you walk through the door. Pretend you are a parent calling for an appointment. Listen to the way phone calls are handled. Is the parent offered a flexible set of potential appointment times to allow for school or childcare schedules? Explore the waiting room. Is there a place for children to play while a parent is waiting for an appointment or meeting with someone?

If you are meeting with a parent at home, anticipate the situation and circumstances. Can you make accommodations in your conversations depending on whether children are present and within hearing distance? If the television is on to occupy or distract the children, are you annoyed by the extra noise? How would you respond? Are you prepared to answer children's questions about why you are there and what you will be doing with their mother or father? If the home is messy or not clean, can you sit comfortably and focus on the conversation? You may have opportunity to see the parent and child interact. If their interaction is not positive or age-appropriate, have you thought about what you might say to guide or role model a better approach without seeming intimidating or threatening? Are you prepared to acknowledge a parent's strengths and capacity to interact well with his or her children?

Considering issues like these, in advance, will help you respond in sensitive, respectful, family-focused ways.

 © 2013 ParentingWell. All rights reserved.

ENGAGE:

Challenge your assumptions

TRY THIS TRUE-FALSE QUIZ:

1. Parenting is a significant life domain for adults with behavioral health conditions.

   ☐ True ☐ False

2. Adults with mental health conditions are as likely as other adults to have children.

   ☐ True ☐ False

3. Their children will definitely have problems.

   ☐ True ☐ False

4. A parental mental health condition alone does not increase the likelihood of child abuse.

   ☐ True ☐ False

5. Parenting may be challenging, but children are often a source of meaning and hope for parents.

   ☐ True ☐ False

SEE BELOW FOR ANSWERS.

 © 2013 ParentingWell. All rights reserved.

THE ANSWERS:

1. **True**. Parenting is a priority for many people, right up there with housing and employment, and they wish to parent well.
2. **True**. Adults living with behavioral health conditions are at least as likely to be parents as individuals without mental health or substance abuse diagnoses. This is true for men as well as women.
3. **False**. Their children will not necessarily have problems. It's not a direct link.

   Yes, some illnesses have a biological connection; inheritance can make a child prone to developing problems. However, children themselves have resources—children can be sturdier or more vulnerable—at different ages and stages, and depending on when and for how long they are "exposed" to a parent's behavioral health condition. They benefit from supports, education about mental health, and help with coping strategies—to develop resilience.

4. **True**. Parents with behavioral health conditions are not necessarily abusive.

   We do know that an accumulation of challenges may increase the likelihood of abuse or neglect—for example, poverty, unemployment, mental health conditions and substance abuse. All parents benefit from supports to overcome adversity.

5. **True**. Yes, raising children presents challenges, but this is true for all parents.

   Many aspire to the typical life goals most of us share—parenthood, family life, etc. Children can be a source of motivation and hope for the future for parents.

 © 2013 ParentingWell. All rights reserved.

WHY ARE THESE ANSWERS IMPORTANT?

- All parents benefit from supports. With the right supports, outcomes for parents and their children can be improved.
- Working with adults living with behavioral health conditions who become parents is the earliest form of prevention of problems in children.
- Parents should be supported and reinforced for seeking help, rather than fearing negative attitudes and stigma from practitioners, or fearing the potential loss of custody that may happen when untested assumptions are made about parents' capacity to do a good enough job.

CHALLENGE YOUR ASSUMPTIONS:

It is critical to examine your own assumptions and opinions about parenting and behavioral health challenges – mental illness and addictions. You have a unique background and history of experiences that have shaped how you view the world. Your family, friends, colleagues, and the professional and social circles in which you spend time inform your worldview. Layered on top of these influences is the impact of the media and how it shapes your perceptions of both parenting and behavioral health. Acknowledging your assumptions may be difficult. Exploring why you feel the way you do and where your ideas come from about certain experiences or groups of people can be challenging, painful and complicated. You may have a parent with mental illness or a substance use issue, for example, and have childhood experiences of providing care for your parent. You may be a parent living with addiction issues and in recovery. You may need support in understanding how your personal experiences of parenting, health and wellbeing affect you today, as a parent, family member or as a practitioner. It may be helpful to connect with a supportive family member, friend, or a peer with similar lived experiences, or with an online or Facebook group as you sort this out.

 © 2013 ParentingWell. All rights reserved.

ENGAGE:
Translating language into feelings

Our culture is infused with violence and our work with parents should be trauma-informed. Many parents with behavioral health challenges have been victims of or witnessed violence in their lives, with traumatic consequences. Without even thinking, people may use language that is potentially triggering for clients who are parents. Parents may use these words and phrases themselves. For example, there are many expressions that come from or may convey a vivid, abusive or even violent impression. Some of these are provided below. Take a few minutes to make a list of common phrases that actually suggest violent or abusive meanings, and then identify alternatives. You may want to suggest these alternatives to the parents you work with, especially to use in conversations with their children.

Examples:

"I could kill you"	This could mean, "I am really angry with you."
"I felt like strangling him."	This could mean, "I was really frustrated by his behavior."

 © 2013 ParentingWell. All rights reserved.

EXPLORE:

Topics for reflection

As you begin to explore parenting and family experiences with the individual who is a parent, issues or themes may come up for you. Some practitioners think they are not well suited to working with parents because they are not parents themselves. However, you have parents or people who served as parents when you were growing up, and you have your own lived experience of family and daily life. It is important to understand how past and current circumstances and situations contribute to the work you do. You can drsw from them to inform your work or recognize issues that come up that you may need to reflect on yourself, with the help of a peer, supervisor or counselor.

Reflect on your heritage.

What is your cultural/racial/ethnic heritage? Are your parents immigrants? Are you? Even if you and your parents were born in this country, traditions are passed down from generation to generation. And different regions of the USA have customs and traditions (e.g., North versus South, or East Coast versus the Mid-West in the USA). What are your family's expectations regarding family life? Do you spend holidays with your parents? What would happen if you didn't? Do you serve traditional foods on particular family occasions? Consider the ways in which your cultural/racial/ethnic heritage influences you in your family life and, possibly, your expectations for others.

How do you define yourself?

Your identity contributes to your behavior, the way you respond to others, and the ways others respond to you. Your identity may shape your priorities, values and attitudes. List the five identities most important to you, for example, as they relate to family roles, relationships, and work. For example, daughter, social worker, significant other, runner, etc. Would others define you in the same way? What do they see when they look at you. There may be things they can't see that really matter to you. Think about how you define the parents you work with. Think about how they define themselves.

 © 2013 ParentingWell. All rights reserved.

Consider your 24-hour day.

Recognition of the complexity of daily life is helpful in understanding a parent's situation and the ways in which his or her behavioral health, family experiences and children interact. One strategy might be to begin a conversation with parents by asking them to describe a typical day. It may be helpful for you to understand the usefulness of this approach by mapping out your own typical day. Take 10 minutes and jot down all the things you do, or are supposed to do, from the time you wake up until you go to bed. (If you are the parent of a baby or young child, you may be up in the nighttime as well!) Hopefully, you will begin to appreciate the complexity of your own life, the contingencies that operate, and the skills it takes to get everything done. Using this approach with parents will help you gain an understanding of their life challenges and demands, as well as help identify the strengths and vulnerabilities they may bring to daily living.

 © 2013 ParentingWell. All rights reserved.

EXPLORE:

Create a positive identity

For individuals living with behavioral health conditions, to be considered a parent is a much more positive identity than to be labeled a patient. Yet many of the unique qualities of an individual can get lost as he or she navigates the service system, treatment and recovery. It may help you to think of questions to pose to parents by asking yourself some of the same questions. Below are some samples to guide a conversation about what is important or meaningful, to begin to define a more positive identify and inspire hope and optimism for change. Try them out yourself and add other questions you think might be helpful. When you have negative thoughts about yourself, try replacing them with positive thoughts drawn from this list, or perhaps with positive statements or affirmations you identify through this exercise.

- How would you describe yourself?
- What do you like about yourself?
- Name three things you do well.
- What kind of food do you like? Music? Television shows?
- Other?

 © 2013 ParentingWell. All rights reserved.

EXPLORE:

Daily activity log

Keep track of the things you do in a typical day or for several days, including eating, napping and sleeping. Think about what you do. Are there activities that take up most of your time? Do you have time to do the things you want to do? Are there times that are more stressful? More relaxing? Are you getting enough sleep? Think about your priorities and what you might want to add or subtract from your daily routine.

12:00 Midnight	12:00 Noon
1:00 AM	1:00 PM
2:00 AM	2:00 PM
3:00 AM	3:00 PM
4:00 AM	4:00 PM
5:00 AM	5:00 PM

 © 2013 ParentingWell. All rights reserved.

6:00 AM	6:00 PM
7:00 AM	7:00 PM
9:00 AM	9:00 PM
10:00 AM	10:00 PM
11:00 AM	11:00 PM

 © 2013 ParentingWell. All rights reserved.

EXPLORE:

Focus on positives

It can be quite a challenge to focus on positives or identify strengths when you're depressed or feeling like nothing is going right. Try keeping a journal of positives or strengths for a week to remind yourself that sometimes things go well and that there are ways in which you are quite capable. These don't have to be big events or super special talents. Quite simply, having a conversation that goes smoothly with your child is a positive event. The ability to organize yourself to fix dinner in the evening is a strength. Try to identify three things each day for a week to see what you notice.

DAY	POSITIVES AND STRENGTHS
**EXAMPLE: ANY DAY**	1. Got to an appointment on time.<br>2. Fixed my daughter a good breakfast.<br>3. I have a good sense of humor.
**SUNDAY**	1.<br>2.<br>3.
**MONDAY**	1.<br>2.<br>3.

 © 2013 ParentingWell. All rights reserved.

**Tuesday**	1.<br>2.<br>3.
**Wednesday**	1.<br>2.<br>3.
**Thursday**	1.<br>2.<br>3.
**Friday**	1.<br>2.<br>3.
**Saturday**	1.<br>2.<br>3.
**Sunday**	1.<br>2.<br>3.

 © 2013 ParentingWell. All rights reserved.

EXPLORE:
Focus on strengths

The following are "worst case" scenarios, in which the practitioner says all the "wrong" things. These are simply to provide examples of strategies that make thinking about strengths difficult. Suggestions for a more positive approach are provided below.

Worst Case Scenarios:

1. MAKING ASSUMPTIONS.

Practitioner says: "So you're homeless because you weren't able to pay the rent?"

Practitioner thinks: You must be one of those homeless people who think the world owes them something.

Practitioner feels: I am so overwhelmed by your problems.

Parent says: "Yeah, the landlord kicked me out.

Practitioner thinks: You won't even take responsibility for the situation. Like, it's the landlord's fault…

Practitioner feels: I can't think of any way to fix them for you.

Actual: The parent has a serious behavioral health condition that requires her to go to many treatment appointments; therefore, she is not able to hold a job or to pay the rent. However, she prioritizes her treatment and manages her illness well.

2. ATTRIBUTING BAD INTENTIONS OR TRAITS.

Practitioner says: "You need to do get your kids off to school on time."

Practitioner thinks: You're not going to 'trick' me or anyone else into taking care of things for you.

 © 2013 ParentingWell. All rights reserved.

Practitioner feels: I have too much of my own work to do. And I'll get in trouble if you don't do a better job of parenting.

Parent says: "I just can't get everything done by the time the bus comes."

Practitioner thinks: You're just too lazy to get out of bed in the morning.

Practitioner feels: There are plenty of days when I don't feel like getting out of bed in the morning.

Actual: The parent takes medication for her depression that makes her quite groggy in the morning. She could stop taking her medication to meet her children's needs, but that would compromise her treatment plan. She is in a bind.

3. DISQUALIFYING OR MINIMIZING WHAT THE OTHER PERSON SAYS.

Practitioner says: "So, you really believe that using drugs today helps you cope with being sexually assaulted by your uncle 20 years ago?"

Practitioner thinks: You would come up with any excuse to use drugs or alcohol.

Practitioner feels: I could use something to help me cope!

Parent says: "When I'm by myself, it's all I think about—what it felt like, what he said to me."

Practitioner thinks: That was 20 years ago—how could that still be such a big deal to you now?

Practitioner feels: You want to hear about problems? You should know what I'm going through!

Actual: The Parent "self-medicates" with drugs and alcohol to ease the pain she feels about her sexual assault. While this is not a healthy strategy, it was available to her when there was no one or nothing else to comfort her. Meanwhile, she has managed to keep herself alive for 20 years.

 © 2013 ParentingWell. All rights reserved.

4. FEELING CRITICIZED BY ANOTHER, OR CRITICIZING THE OTHER PERSON'S POINT OF VIEW.

Practitioner says: "You say your boyfriend takes your food stamps and sells them to buy drugs..."

Practitioner thinks: You must think I'm stupid if you think I believe that!

Practitioner feels: No one appreciates the work I do.

Parent says: "I give them to him so he'll let me use the car."

Practitioner thinks: Well that's really stupid, to give up food in exchange for a car ride.

Practitioner feels: Why do these parents keep making bad decisions? It's so frustrating.

Actual: The parent needs her boyfriend's car to visit regularly with her children who are in foster care. She understands that her children need her to keep her commitment to them.

 © 2013 ParentingWell. All rights reserved.

Strategies for Encouraging the Identification of Strengths:

1. ACKNOWLEDGING OR VALIDATING WHAT THE OTHER PERSON TELLS YOU.

Example: *"What happened to you was wrong and painful, but you were very brave to 'hang in' there."*

2. WANTING TO KNOW MORE.

Example: *"What did you do then to solve the problem?…That was pretty resourceful of you!"*

3. GETTING SPECIFIC.

Example: *"Well, you say you're not smart enough to get your GED. What makes you feel that you're not smart?…But in a different situation, you do very well!"*

4. ENCOURAGING THE OTHER PERSON TO COMMUNICATE IN ANY WAY POSSIBLE.

Example: *"Look, I know it's hard for you to talk about this, but maybe you could take this little notebook and write down things you feel good about as you notice them during the day."*

 © 2013 ParentingWell. All rights reserved.

ParentingWell® STRENGTHS & GOALS					
Here is a list of things you may need to do as a parent. For each one that applies to you, *circle* the answer that describes you best.	This is a strength of mine.	I do this okay.	I'd like to do this better.	Does not apply.	Check items to work on.
1. Manage everyday household tasks	Strength	Okay	Better	DNA	
2. Plan and make healthy meals	Strength	Okay	Better	DNA	
3. Understand the relationship between my feelings and my actions	Strength	Okay	Better	DNA	
4. Manage my family's money	Strength	Okay	Better	DNA	
5. Set limits with my child	Strength	Okay	Better	DNA	
6. Have positive interactions/visits with my child	Strength	Okay	Better	DNA	
7. Have a pleasant routine with my child	Strength	Okay	Better	DNA	
8. Find fun things to do with my child	Strength	Okay	Better	DNA	
9. Get adequate child care for my child	Strength	Okay	Better	DNA	
10. Balance work or school, and parenting	Strength	Okay	Better	DNA	
11. Know what to do when my child has problems	Strength	Okay	Better	DNA	
12. Identify my child's strengths	Strength	Okay	Better	DNA	
13. Have positive "family time"	Strength	Okay	Better	DNA	
14. Know my legal options as a parent	Strength	Okay	Better	DNA	
15. Get help for myself, if I need it	Strength	Okay	Better	DNA	
16. Talk with my child about my situation or worries	Strength	Okay	Better	DNA	
17. Keep in touch with my child who is not living with me	Strength	Okay	Better	DNA	
18. Live a substance free lifestyle	Strength	Okay	Better	DNA	
19. Communicate well with my child	Strength	Okay	Better	DNA	
20. Have good relationships with my child's caregivers/helpers	Strength	Okay	Better	DNA	
21. Express anger without hurting anyone	Strength	Okay	Better	DNA	
22. Keep my child and myself safe	Strength	Okay	Better	DNA	
23. Make time to take care of myself	Strength	Okay	Better	DNA	
24. Manage stress and worries in healthy ways	Strength	Okay	Better	DNA	
25. Cope with bad things that have happened to me in my life	Strength	Okay	Better	DNA	
26. Get special services and supports for my child	Strength	Okay	Better	DNA	
27. Other:	Strength	Okay	Better	DNA	

©2003 J. Nicholson, PhD & A. Henry, ScD, OTR/L
ParentingWell® is a trade mark of Strengths Based Solutions, LLC
www.parentingwell.org

PLAN:

Helping parents evaluate options

Parents living with behavioral health conditions may benefit from support in identifying and evaluating options to make informed decisions. You can provide them with information about possibilities that exist that they may not be aware of, for example, community resources or entitlements. You might give an example of a decision-making strategy by making a list of possible options, and writing down positive and negative effects, or pros and cons. Walk the parent through the process, in a clear and concrete way. “If you choose this, then this will happen.” “If you choose that, then you might miss out on this.” In this way, you help the parent assess the options and make a choice about how to proceed.

Your role is to help parents see choices where they may not have been clear before. For example, a mother recently arriving at a domestic violence shelter may believe that her only option to support her family is to return to the partner who abuses her. A practitioner can help this mother think about what her goals are, and what options exist to help her achieve new goals successfully. This is where having good, up-to-date information about resources may be quite helpful.

A parent’s priorities may not be what you might suggest to them. You may be challenged in having to accept some choices parents may make and, while you may not be in a position to tell a parent what to do, you can offer realistic, non-judgmental feedback on the potential implications or consequences of a particular choice. This is not an easy task. It can be difficult to separate out your own opinions about the choices people make from their opinions. You can provide respectful feedback if their choices have negative consequences—not “I told you so,” but “What can we do together now to make this better?” You cannot sit by while individuals make choices that put themselves or others in danger or at risk. This is one time when you must back up your opinion with a clear statement of the potential consequences, especially if it involves you having to notify child welfare officials or the police. Parents are encouraged to seek

 © 2013 ParentingWell. All rights reserved.

additional professional help, if it seems warranted (e.g., children’s mental health services). If the need for child welfare services becomes evident, then you can act as an ally in collaborating with the parent to obtain help.

The key to these tasks is creating opportunities for success. Success may be defined differently by different people. It may be something very big, or something that others might think is small but that feels big to the parent. Making and keeping a dentist appointment, while it might seem like a small success, may feel huge to a mother who hasn’t been able to find the time and energy to take care of herself. Other successes, like finding housing, or ending an abusive relationship, or finding a job—are more likely to be viewed as huge by others. Benchmarks should be set along the way to mark progress and to set a time to re-evaluate and make a new plan, if necessary. Each success, small or large, represents considerable effort on the part of the parent, and should be celebrated as a step towards recovery.

 © 2013 ParentingWell. All rights reserved.

PLAN:

Helping parents make choices

Many parents living with behavioral health conditions may have been in situations where others always told them what to do, or they may have made decisions that were "wrong" or had bad outcomes. They may need practice coming up with alternatives and thinking through what the potential consequences might be. They may need information about possibilities that exist that they may not be aware of, for example, community resources or entitlements. You can give an example of a decision-making strategy by making a list of possible options, and writing down positive and negative effects, or pros and cons. Walk the parent through the process, in a clear and concrete way. "If you choose this, then this will happen." "If you choose that, then you might miss out on this." In this way, you help the parent assess the options and make a choice about how to proceed.

Your role is to help parents see choices where they may not have been clear before. For example, the mother recently arriving at a domestic violence shelter may believe that her only option to support her family is to return to the partner who abuses her. A practitioner can help this mother think about what her goals are, and what options exist to help her achieve new goals successfully. This is where having good, up-to-date information about resources may be quite helpful.

A parent's priorities may not be what you might suggest to them. You may be challenged in having to accept some choices parents may make and, while you may not be in a position to tell a parent what to do, you can offer realistic, non-judgmental feedback on the potential implications or consequences of a particular choice. This is not an easy task. It can be difficult to separate out your own opinions about the choices people make from their opinions. You can provide respectful feedback if their choices have negative consequences—not "I told you so," but "What can we do together now to make this better?" You cannot sit by while individuals make choices that put themselves or others in danger or at risk. This is one time when you must back up your opinion with a clear statement of the potential consequences, especially if it involves

 © 2013 ParentingWell. All rights reserved.

you having to notify child welfare officials or the police. Parents are encouraged to seek additional professional help, if it seems warranted (e.g., children's mental health services). If the need for child welfare services becomes evident, then you can act as an ally in collaborating with the parent to obtain help.

The key to these tasks is creating opportunities for success. Success may be defined differently by different people. It may be something very big, or something that others might think is small but that feels big to the parent. Making and keeping a dentist appointment, while it might seem like a small success, may feel huge to a mother who hasn't been able to find the time and energy to take care of herself. Other successes, like finding housing, or ending an abusive relationship, or finding a job—are more likely to be viewed as huge by others. Benchmarks should be set along the way to mark progress and to set a time to re-evaluate and make a new plan, if necessary. Each success, small or large, represents considerable effort on the part of the parent, and should be celebrated as a step towards recovery.

 © 2013 ParentingWell. All rights reserved.

PLAN:
Back-up childcare

This activity will help you develop a plan of action for obtaining childcare in the event that you are unable to care for your children for any reason. You should work on this plan when you can take the time to think clearly about the best strategies to meet the needs of you and your family. Developing a plan during a time of calm—not a time of crisis—will go a long way toward you and your family being confident that you are prepared to manage any unexpected situations where you'll need assistance with childcare.

It might take a while for you to gather this information, but it will be worth it to provide you and your family with a blueprint for coping during a difficult time. It is important to make this a real working plan of action, to involve those you will be listing in creating the plan. You want to know if they can be counted on to help you and where they can be reached at all times.

Once you have completed the plan, you should discuss it with your children if they are old enough and your partner, relatives, or those you live with, so they can put it into action when necessary. Post a copy where it's easy to find, and make a point of updating it every few months.

 © 2013 ParentingWell. All rights reserved.

This back-up plan is for (child/children's names and dates of birth):

If my child/children need(s) to miss time at school or from other activities, please contact:

	NAME	PHONE
**Childcare provider/School:**		
**Childcare provider/School:**		
**After-school program/Activity:**		
**After-school program/Activity:**		
**Other:**		
**Other:**		
**Other:**		

CONTINUED ON THE NEXT PAGE

 © 2013 ParentingWell. All rights reserved.

For back-up childcare for a few hours, contact:

NAME	ADDRESS	PHONE

For back-up childcare for overnight, contact:

NAME	ADDRESS	PHONE

For long-term back-up childcare or respite (like a weekend or week), contact:

NAME	ADDRESS	PHONE

 © 2013 ParentingWell. All rights reserved.

PLAN:
The importance of self-care

Self-care is a good example of a priority that is hard to set for many people. You may need to work together with the parent to understand and embrace the reality that parents have to take care of themselves to be able to provide care for their children. It may be instinct for a parent to respond to a child's needs first. Coupled with the guilt and shame some parents may feel about their behavioral health conditions and the impact of their illnesses on their children, parents may feel twice as guilty if they think they are ignoring their children to take care of their own needs. Unfortunately, parents may prioritize their children's needs in the short-term, and put themselves at risk over time, if they become "run down" or have decreased capacity to cope with day-to-day challenges. An effective, positive self-care strategy helps us "recharge our batteries" when our energy is running low.

Self-care strategies contribute to coping skills and resiliency. A self-care strategy may help you to feel better when you are hurt or feeling bad. Self-care is also something you can do in an ongoing way, so you can deal better with stress and crises as they come up. People who take good care of themselves are likely to be more resilient, to cope better and to have better outcomes when facing challenges. Self-care strategies are individualized. What is effective for one person will not necessarily work for another. What is important is to identify what works best for you or, in the ParentingWell practice approach, the parent you're working with.

 © 2013 ParentingWell. All rights reserved.

PLAN:

Developing positive self-care strategies

Taking care of yourself is one of the most important things you can do as a parent. You really need to know yourself to take care of yourself—to establish a routine, to know when you might be stressed, and to develop effective self-care strategies to build into your daily life. Parents often have to work hard to find the time and energy to do something for themselves, which may not be their priority on any given, busy day. You may feel guilty or even neglectful of your children in the process of taking time for yourself—many parents do. Identify what works best for you—something that doesn't conflict with your children's needs or their safety—and that helps you feel and stay well.

A self-care strategy helps you feel better. What works for one person does not necessarily work for another. At the same time, not all strategies for helping us feel better are healthy or safe. While everyone has bad habits, your goal may be to shift from negative strategies to more positive strategies over time.

Positive strategies add to your health and well-being, not jeopardize it. Some examples of everyday activities that may help you feel better are reading for pleasure, exercising, listening to music, dancing, playing with a pet, trying a new recipe or participating in a faith community. Building positive strategies into your daily routine shows your children you are taking care of yourself. You can help your children discover healthy ways to take care of themselves, too. Take a minute to think about what positive self-care strategies help you feel better and exactly how they make you feel.

 © 2013 ParentingWell. All rights reserved.

POSITIVE SELF CARE STRATEGY	THIS MAKES ME FEEL

Keep this list where it's easy to find. Look at it every day to remind yourself to take a moment for yourself. Remind yourself of what helps when you are feeling anxious or stressed. Share it with family and friends, so they can help you remember to take good care of yourself. If your children are old enough, sit with them to help them make a self care list of their own.

 © 2013 ParentingWell. All rights reserved.

PLAN:

Creating a self-care plan

Taking care of yourself helps you take better care of your children. Making time for yourself can be difficult, but it is an investment in your wellbeing. You are serving as a great role model for your children! Encourage them to learn about the things that help them feel well, too. Once you've identified your positive care strategies (try using the Developing Positive Self Care Strategies activity), use the grid below to create a personalized self-care plan to remind you of what strategies are useful and how to make them happen. Keep this plan where it's easy to find and look at it, especially when you are anxious or stressed. Share it with family and friends. They can help you remember to take good care of yourself.

 © 2013 ParentingWell. All rights reserved.

SELF-CARE STRATEGY	HOW DOES THIS MAKE ME FEEL?	HOW OFTEN DO I DO THIS?	WHAT KEEPS ME FROM DOING IT MORE OFTEN?	HOW CAN I DO THIS MORE OFTEN?
Example: exercise	*Energized, good about my body, healthy*	*Once a week*	*No childcare*	*Leave the kids with their grandmother*

 © 2013 ParentingWell. All rights reserved.

PLAN:

Dealing with your own stress

Stress management is an important part of avoiding burn-out, especially when you are working with parents and families dealing with many challenges or living in adverse conditions. You can search online and easily find examples of strategies for stress management. Here are three examples you can try out for yourself or with the parents you see.

IDENTIFY PRIORITIES TO MAKE A CHANGE.

Think about your own priorities. Envision a change you might like to make—perhaps find a new apartment or take an advanced course in motivational interviewing. Think through the steps it will take (e.g., check Craig's list, ask friends if they know of anything, etc.). Write down examples of action steps, identify who might help you take the step or what other resources you may need to take the next step. Set reasonable deadlines and review your progress periodically to add new steps, revise steps and add new deadlines. Celebrate each step along the way and the final achievement of your goal.

ADJUST YOUR PLAN TO ACHIEVE YOUR GOAL.

Feelings of anxiety and stress may be a sign that you are not taking good care of yourself. Oftentimes, these feeling can happen when you are not able to do what you want to do, or when you feel like you have failed at an important task. One reason this may happen is that we may set ourselves up for failure by setting goals that are too difficult to achieve. One strategy to help you address feelings of anxiety and stress is to modify or reframe professional and personal tasks to make them more achievable. For example, a challenging professional goal may be to complete a master's degree this year while you are working full-time at the agency. You might need to modify your timeframe or cut back on your work hours to set a more reasonable, do-able goal. Instead of saying "I'm going to run a marathon at the end of one month of exercising," a more achievable goal might be to say, "I'm going to run as far as I can, hopefully two

 © 2013 ParentingWell. All rights reserved.

miles, at the end of one month of exercising." Remember, creating opportunities for your own success is just as important as helping the parents you work with be successful.

MAKE SELF-CARE A PRIORITY.

Generate a list of activities that help you feel better and identify how they make you feel. Some examples are provided below. Include activities you find particularly helpful when you want to relax.

POSITIVE SELF CARE STRATEGY	THIS HELPS ME FEEL
doing a crossword puzzle	relaxed, calm, smart
exercise	energized, good about my body, healthy
participate in a religious activity	spiritual, involved in a community, centered

 © 2013 ParentingWell. All rights reserved.

PLAN:

A collaborative mapping exercise[1]

ORGANIZING VISION FOR YOU WITH YOUR FAMILY
What would you like the future to look like for you with your children? (Both the near future and the distant future) What does this picture look like?

OBSTACLES	SUPPORTS
What gets in the way?	*What helps you get there?*

PLAN
What needs to happen next? Lay out some action steps!

[1] Adapted from Madsen, W.C. & Gillespie, K. (2014) Collaborative helping: A strengths framework for home-based services. NY: John Wiley & Sons, Inc.

© 2013 ParentingWell. All rights reserved.

ACCESS & ADVOCATE:
Consider your own support network

Relationships that are truly supportive may be two-way streets or, in some cases, you may be giver or the recipient of help or advice. Family members or friends may have expectations for you, or you of them, regarding how they can help and what, if anything, they may expect in return. Think about a recent problem, challenge, or need you may have faced. It can be anything from watching your dog while you go away for the weekend, to driving you to a doctor’s appointment, to loaning you money to replace your laptop computer. Different challenges require different solutions, and a different give-and-take in relationships. Try responding to these:

- Make a list of the family members and friends you can count on if you need help.
- How and when can they help you? Next to each name, make some notes about their situations, and what and when you might ask for help. Different needs may require different types of support from your network—not everyone may be right for each situation.
- Which family members or friends might ask for or need something in return? What might you think to do for them? Would you feel grateful for their help or feel guilty, as if you were a burden?
- Relationships are not solely based on helping each other. How do you maintain your relationships in other ways besides helping? What can you do to balance your relationships? Is a balance of give-and-take important?

 © 2013 ParentingWell. All rights reserved.

ACCESS & ADVOCATE:

Communicating with children of different ages

Communicating with children differs a lot depending on their ages and stages and your skills and preferences—and it's not always about talking. Here are some suggestions for communicating with your children. Space is provided to check off your preferences and to add others we haven't thought of. How does your child communicate with you?

0 – 2 YEARS OLD	5 – 12 YEARS OLD
☐ Showing love & affection	☐ Being concrete
☐ Holding her	☐ Using her words
☐ Talking to him	☐ Getting down to his level, like on the floor
☐ Gentle touching	☐ Talking about feelings
☐ Rocking	☐ Singing songs
☐ Cuddling	☐ Telling stories
☐ Singing	☐ Reading books
☐ Imitating sounds she makes	☐ Playing games
☐ Being there when I can	☐ Going for rides in the car
☐ ________________________	☐ Eating meals together
☐ ________________________	☐ ________________________

 © 2013 ParentingWell. All rights reserved.

2 – 5 YEARS OLD	13 - 18 YEARS OLD
☐ Beginning to label feelings	☐ Talking about feelings
☐ Drawing pictures	☐ Discussing things honestly
☐ Going for walks	☐ Keeping the door to communication open
☐ Providing reassurance	☐ Providing things to read
☐ Singing songs	☐ Texting on a cell phone
☐ Telling stories	☐ Being available
☐ Explaining things in simple words	☐ Talking on her timetable, when she is ready
☐ ________________________	☐ Respecting his growing independence
	☐ ________________________

ADULT CHILDREN	
☐ Treating her like an adult	☐ Talking about feelings
☐ Discussing your situation openly, and working together to communicate with the next generation	☐ Meeting together with your doctor, therapist or case manager
☐ Asking and answering questions	☐ __________________________
	☐ __________________________
	☐ __________________________

 © 2013 ParentingWell. All rights reserved.

ACCESS & ADVOCATE:
Communicating about your behavioral health condition

Below you will find a list of pros and cons to communicating with your children and others about your behavioral health condition. These are just a few. Some may describe how you feel and some may not. Check off the ones that apply to you. Additional space is provided for you to enter your own ideas. Once you have made your own list, consider the questions below. This exercise should help you answer these questions about communicating with your children and others about your situation.

PROS	CONS
☐ I might feel less blamed.	☐ I might feel more shame or guilt
☐ If people understand me better they might have more compassion.	☐ Others may show compassion without truly understanding. I don't want pity.
☐ It would give people the opportunity to help me.	☐ My children may feel burdened by knowing I have an illness.
☐ Providing accurate information breaks down stereotypes and negative attitudes about behavioral health conditions.	☐ Hiding my behavioral health condition leaves me less vulnerable to stereotypes and stigma.
☐ I could make a plan for my children with family members for when I'm not doing well.	☐ People will be afraid of me, or for me, if they think I'm nuts.
☐ It would reduce my children's fear or guilt.	☐ My children might feel responsible.

 | © 2013 ParentingWell. All rights reserved.

PROS	CONS
☐ ______________________	☐ ______________________
☐ ______________________	☐ ______________________
☐ ______________________	☐ ______________________

 © 2013 ParentingWell. All rights reserved.

ACCESS & ADVOCATE:

Your network of family and friends

Your family and friends are important for many reasons—from having fun together to helping each other out. Supportive relationships may be two-way streets or, in some cases, you may simply give or receive help or advice. Family members or friends may have expectations for you or you for them, about what they can do and what, if anything, they may expect you to do in return. Make a list of family and friends you can count on when you need help or when you just want to have fun.

FAMILY & FRIENDS:<br>NAME	DESCRIBE THE RELATIONSHIP: FUN? HELP? DEPEND ON? TAKE CARE OF?	PROS/BENEFITS/ HOW CAN THEY HELP?	CONS/CHALLENGES/ WHAT DO THEY EXPECT IN RETURN?

 © 2013 ParentingWell. All rights reserved.

Contact Us

Joanne Nicholson, PhD
Professor, Institute for Behavioral Health
Heller School for Social Policy and Management at Brandeis University
Waltham, Massachusetts
jnicholson@brandeis.edu
heller.brandeis.edu/parents-with-disabilities/index.html

Kelly English, PhD, LICSW
Director, Children's Behavioral Health Knowledge Center
Massachusetts Department of Mental Health
Boston, Massachusetts
kelly.english@state.ma.us
www.cbhknowledge.center

Recommended Citation

Nicholson, J. & English, K. (2019). *ParentingWell Workbook*. Boston, MA: Massachusetts Department of Mental Health & National Research Center for Parents with Disabilities. https://www.cbhknowledge.center/parentingwell-resources

© ParentingWell 2019

Funded by

This work is funded by the Massachusetts Department of Mental Health and a grant from the National Institute on Disability, Independent Living, and Rehabilitation Research (grant #90DPGE0001-01-01) as part of the National Research Center on Parents with Disabilities at Brandeis University. The statements presented in this publication are solely the responsibility of the authors and do not represent the views of the federal agency.

National Research Center for Parents with Disabilities
Parents Empowering Parents
Padres Empoderando a Padres

Mass DMH
Department of Mental Health

CBH KNOWLEDGE CENTER
