# Supplementary material for: The ParentingWell Practice Approach: Adaptation of Let’s Talk About Children for Parents With Mental Illness in Adult Mental Health Services in the United States
Source: Front Psychiatry. 2022 Apr 7;13:801065. doi: 10.3389/fpsyt.2022.801065 (PMC9021592; doi:10.3389/fpsyt.2022.801065)
Supplement: Supplementary file 3 [file Data_Sheet_3.PDF]

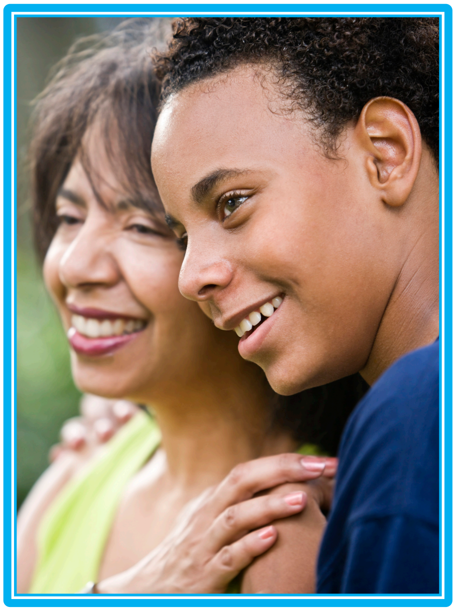

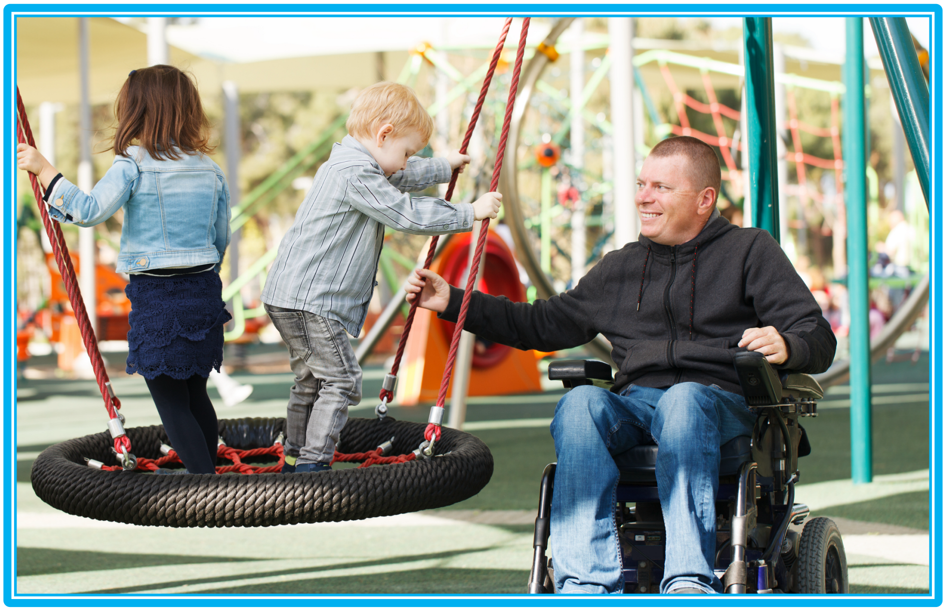

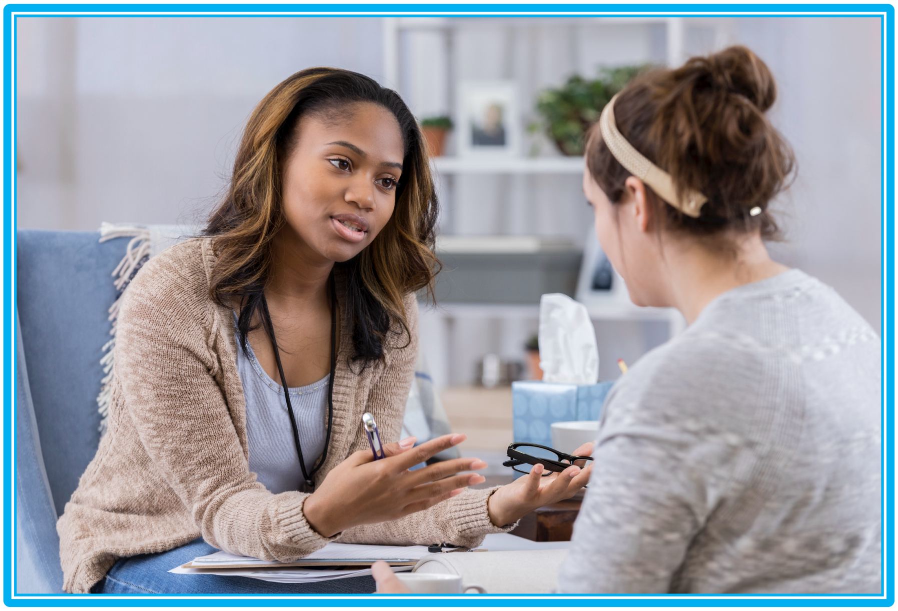

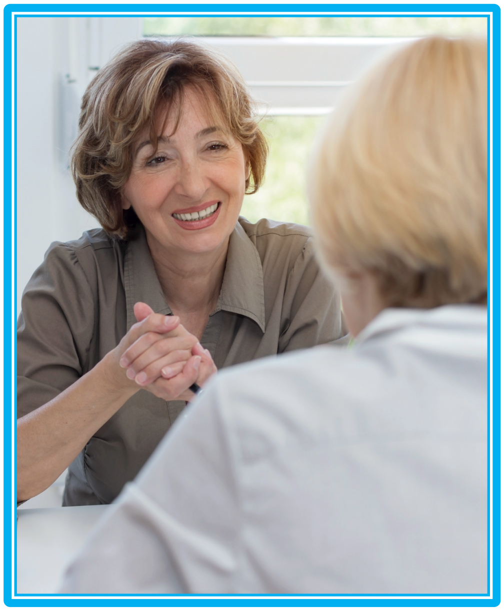

ParentingWell Self-Assessment & Supervisory Tools

Nicholson, J. & English, K. (2019). ParentingWell Self-Assessment & Supervisory Tools. Waltham, MA: National Research Center for Parents with Disabilities, Brandeis University and the MA Department of Mental Health.
https://www.cbhknowledge.center/parentingwell-resources © ParentingWell 2019

ParentingWell Practice Profile - ENGAGE – Self Assessment

This worksheet is for staff to assess their own practice with respect to parenting practice as defined by the ParentingWell Practice Profile. This worksheet is to be used in preparation for meeting with your supervisor. Use the *Engage* Core Element of the Practice Profile to identify both the strengths and areas needing improvement in your practice. Using a copy of the Engage Core Element from the practice profile, read the description of the Core Element all the way through so you can get a sense of what the practice looks like as a "whole." Make note (you may highlight, underline or circle items on your copy of the profile) of specific items that reflect your current practice, then assess your practice for each sub-category using a rating scale of 1 to 10 as defined below. Record your rating for each sub-category and provide examples of strengths and areas needing improvement on this worksheet.

10	Ideal practice proficiency	My practice in this area demonstrates a consistently high degree of mastery in a wide range of situations and with all persons served.
5	Developmental practice proficiency	My practice in this area demonstrates a good understanding and skill level. My practice is strong, but only in some situations and with some persons served.
1	Unsatisfactory practice proficiency	This is a new or emerging skill for me. It is not yet present in my practice.
0	N/A	This practice is not part of my responsibilities.

© ParentingWell 2019

ENGAGE	Rating	Notes/Examples/Questions/New Goals
a. Manage first contacts and ask about parenting status, family composition and relationships.		
b. Provide rationale for talking about parenting and family life.		
c. Respect parent's priorities and goals.		
d. Obtain information about children – where living, who caring for them (if minors), custody, visitation, frequency and extent of contact.		
e. Discuss the relationship between family life and recovery.		
f. Recognize and respond to parent's feelings and concerns, including possible reluctance to discuss family issues.		
g. Reflect on personal lived experience of parenting, family life, culture and community.		

© ParentingWell 2019

ENGAGE	Rating	Notes/Examples/Questions/New Goals
h. Provide respectful, non-judgmental responses to parent's expression of challenges.		
i. Consider own assumptions about behavioral health conditions and parenting.		
j. Recognize that person may have had prior negative experiences in family life and with helping professionals.		

© ParentingWell 2019

ParentingWell Practice Profile - EXPLORE – Self Assessment

This worksheet is for staff to assess their own practice with respect to parenting practice as defined by the ParentingWell Practice Profile. This worksheet is to be used in preparation for meeting with your supervisor. Use the *Explore* Core Element of the Practice Profile to identify both the strengths and areas needing improvement in your practice. Using a copy of the Explore Core Element from the practice profile, read the description of the Core Element all the way through so you can get a sense of what the practice looks like as a "whole." Make note (you may highlight, underline or circle items on your copy of the profile) of specific items that reflect your current practice, then assess your practice for each sub-category using a rating scale of 1 to 10 as defined below. Record your rating for each sub-category and provide examples of strengths and areas needing improvement on this worksheet.

10	Ideal practice proficiency	My practice in this area demonstrates a consistently high degree of mastery in a wide range of situations and with all persons served.
5	Developmental practice proficiency	My practice in this area demonstrates a good understanding and skill level. My practice is strong, but only in some situations and with some persons served.
1	Unsatisfactory practice proficiency	This is a new or emerging skill for me. It is not yet present in my practice.
0	N/A	This practice is not part of my responsibilities.

© ParentingWell 2019

EXPLORE	Rating	Notes/Examples/Questions/New Goals
a. Provide framework and support for talking about how things are going with children and family.		
b. Discuss daily routines, household management and child care.		
c. Listen carefully, with genuine curiosity.		
d. Note children's situations, development and functioning.		
e. Guide and provide information about behavioral health, parenting and children's wellbeing.		
f. If parent is separated or disengaged from children, ask about why/how this happened, and about relationships and coping.		
g. Ask about stressful life events and trauma, at the parent's pace.		

© ParentingWell 2019

EXPLORE	Rating	Notes/Examples/Questions/New Goals
h. Discuss cultural norms, family beliefs, and expectations regarding parenting and family life, behavioral health and treatment		
i. Discuss children's understanding of parent's behavioral health condition and how parent communicates about this.		
j. View adult in terms of roles, responsibilities and priorities as a parent and family member.		
k. Consider cultural context and identity as they relate to parent's experiences, strengths, supports and resources.		
l. Assist parent in identifying and building on strengths.		
m. Consider own issues that may be triggered in conversation with parent.		

© ParentingWell 2019

ParentingWell Practice Profile - PLAN – Self Assessment

This worksheet is for staff to assess their own practice with respect to parenting practice as defined by the ParentingWell Practice Profile. This worksheet is to be used in preparation for meeting with your supervisor. Use the *Plan* Core Element of the Practice Profile to identify both the strengths and areas needing improvement in your practice. Using a copy of the Plan Core Element from the practice profile, read the description of the Core Element all the way through so you can get a sense of what the practice looks like as a "whole." Make note (you may highlight, underline or circle items on your copy of the profile) of specific items that reflect your current practice, then assess your practice for each sub-category using a rating scale of 1 to 10 as defined below. Record your rating for each sub-category and provide examples of strengths and areas needing improvement on this worksheet.

10	Ideal practice proficiency	My practice in this area demonstrates a consistently high degree of mastery in a wide range of situations and with all persons served.
5	Developmental practice proficiency	My practice in this area demonstrates a good understanding and skill level. My practice is strong, but only in some situations and with some persons served.
1	Unsatisfactory practice proficiency	This is a new or emerging skill for me. It is not yet present in my practice.
0	N/A	This practice is not part of my responsibilities.

© ParentingWell 2019

PLAN	Rating	Notes/Examples/Questions/New Goals
a. Help parents/parents-to-be identify what they want to change and picture the outcomes.		
b. Assist parents in setting priorities.		
c. Help parents identify options and consider the benefits or costs of choosing one option over another.		
d. Work with parents to set “SMART” goals.		
e. Discuss the value of following up on implementing the plan, evaluating, and revising or setting a new goal to move forward.		

© ParentingWell 2019

PLAN	Rating	Notes/Examples/Questions/New Goals
f. Assist with a problem-solving approach is parents cannot “put the pieces in place” to take steps forward.		
g. Debrief with parents when goals are not achieved to discuss options, to make a new plan, and to use lessons learned to move forward.		
h. Help parents anticipate crises and address vulnerabilities through preventive action or activating resources.		
i. Provide concrete suggestions for time management and other organizational skills.		
j. Assist parent to develop strategies for keeping in touch with children when they are apart.		

© ParentingWell 2019

PLAN	Rating	Notes/Examples/Questions/New Goals
k. Help parents make a back-up plan for providing for children if parents are unavailable.		
l. Provides rationale for taking care of oneself.		
m. Check-in regularly to help parents evaluate progress and adjust action steps if necessary.		
n. Support parents in identifying ways to celebrate small steps and large accomplishments.		
o. Understand that parents' attitudes and beliefs, priorities and actions may not be consistent with one's own.		

© ParentingWell 2019

ParentingWell Practice Profile – ACCESS & ADVOCATE – Self Assessment

Name: ____ **Date of Self-Assessment:** ________________

This worksheet is for staff to assess their own practice with respect to parenting practice as defined by the ParentingWell Practice Profile. This worksheet is to be used in preparation for meeting with your supervisor. Use the *Access & Advocate* Core Element of the Practice Profile to identify both the strengths and areas needing improvement in your practice. Using a copy of the Access & Advocate Core Element from the practice profile, read the description of the Core Element all the way through so you can get a sense of what the practice looks like as a "whole." Make note (you may highlight, underline or circle items on your copy of the profile) of specific items that reflect your current practice, then assess your practice for each sub-category using a rating scale of 1 to 10 as defined below. Record your rating for each sub-category and provide examples of strengths and areas needing improvement on this worksheet.

10	Ideal practice proficiency	My practice in this area demonstrates a consistently high degree of mastery in a wide range of situations and with all persons served.
5	Developmental practice proficiency	My practice in this area demonstrates a good understanding and skill level. My practice is strong, but only in some situations and with some persons served.
1	Unsatisfactory practice proficiency	This is a new or emerging skill for me. It is not yet present in my practice.
0	N/A	This practice is not part of my responsibilities.

© ParentingWell 2019

ACCESS & ADVOCATE	Rating	Notes/Examples/Questions/New Goals
a. Begin the conversation about current supports and resources – emotional, financial, instrumental, professional and personal – that benefit the parent, the child, and the family.		
b. Provide the rationale for the benefits of social support and positive interactions as they contribute to recovery and resilience for both parents and children.		
c. Suggest the benefits of and support parents in helping others as well as asking for help, as mutual give-and-take builds more supportive relationships.		
d. Explore any hesitancy on the part of parents to connect or communicate with others regarding parenting or children, especially to ask for help.		
e. Ask who helps most with child caregiving, if relevant. Who do parents ask for advice about parenting and relationships with children (of any age)?		

© ParentingWell 2019

ACCESS & ADVOCATE	Rating	Notes/Examples/Questions/New Goals
f. Ask parents about strengths of partners or other significant family members, especially those who may be involved as caregivers.		
g. Ask how often and in what context parents interact with other adults (e.g., friends, neighbors, family members), especially other adults who are parents.		
h. Assist parents in identifying individuals or family members who can or do serve as positive role models, or who provide positive assistance to the parent and family.		
i. Ask about resources available or used in the neighborhood and community.		
j. Ask about involvement with other systems, services or practitioners.		
k. Support parents in influencing people and environments that promote recovery for themselves and resiliency for their children.		

© ParentingWell 2019

ACCESS & ADVOCATE	Rating	Notes/Examples/Questions/New Goals
l. Encourage, role model, coach and offer parents opportunities to rehearse and practice interacting with others.		
m. Help parent identify alternative or new social support resources.		
n. Research available opportunities for social support with the parent.		
o. Assist parents in identifying professional sources of support.		
p. Support parents' developing skill, growing confidence in interpersonal situations, maintaining social support networks, and accessing essential resources.		

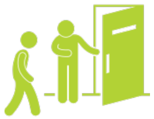

© ParentingWell 2019

ParentingWell

ParentingWell Practice Profile
Individual Skill Development Plan

Name: ____ Position/ Title: _________________

Supervisor: ____

Plan Period: From____ /____ /____ To ____ /____/____

Practitioners providing ParentingWell services need a thorough and continuously improving understanding of the practices that have been identified in the **ParentingWell Practice Profile** as the core elements of the work. Working together with their supervisors, practitioners identify and prioritize the skills that they need to improve and create a plan to strengthen those skills. This **Individual Skill Development Plan** is used to document the steps that will be taken to improve the worker's practices.

Step 1: The practitioner and supervisor use the practitioner's self-assessment to identify and prioritize the skills to be developed. These are called the "Learning Needs." There may be more than one Learning Need.

Step 2: The supervisor and practitioner should create a specific plan to improve the practitioner's skill in the selected areas. The plan might include training, additional or refocused supervision, behavioral rehearsals, being observed by the supervisor, observing a peer, or other activities. Key dates, such as when the plan will start and be completed, should be determined.

Step 3: The supervisor and practitioner write the Learning Needs, Learning Plans, and Key Dates on this form in order to track progress.

Step 4: The supervisor and practitioner share responsibility for implementing this ISD Plan and reviewing progress on the plan at agreed upon intervals. Once it is completed, they discuss whether the plan was successful, document the outcomes in the last section of the plan, and decide on next steps, which may include a new ISD plan or other follow-up.

© ParentingWell 2019

**Learning Need #1** *(What PARENTINGWELL practice element needs to be developed or improved?):*
**Learning Plan #1** *(What activity will occur to help the practitioner develop or improve this skill?):*
**Key Dates #1**<br><br>Start date: ___/___/___ Expected completion date: ___/___/___ Actual completion date ___/___/___

**Learning Need #2** *(What PARENTINGWELL practice element needs to be developed or improved?):*
**Learning Plan #2** *(What activity will occur to help the practitioner develop or improve this skill?):*
**Key Dates #2**<br><br>Start date: ___/___/___ Expected completion date: ___/___/___ Actual completion date ___/___/___

© ParentingWell 2019

**Learning Need #3** *(What PARENTINGWELL practice element needs to be developed or improved?):*
**Learning Plan #3** *(What activity will occur to help the practitioner develop or improve this skill?):*
**Key Dates #3**<br><br>Start date: ___/___/___ Expected completion date: ___/___/___ Actual completion date ___/___/___

Signatures *(after developing the initial plan)***:**

	Signature	Date
**Practitioner**		
**Supervisor**		

© ParentingWell 2019

After the ISD Plan is implemented and completed, describe the outcomes (e.g., activities completed, progress in developing or improving competencies, learning needs that remain):

**Learning Need #1**

**Learning Need #2**

**Learning Need #3**

	**Signature**	**Date**
**Practitioner**		
**Supervisor**		

 © ParentingWell 2019

ParentingWell

Monthly Supervision Guided by Staff Self-Assessment

Definition

Supervision meetings that are guided by the PARENTINGWELL Practice Profile self-assessment are intended to be discussions about the quality and consistency of PARENTINGWELL staff's practice across cases, within and across Practice Profile elements. While every supervisory encounter aims to build upon staff strengths and to increase competency in areas of need, it is important to periodically step away from a focus on the person served, and to focus specifically on the supervisee's own professional development. The supervisor and supervisee have a joint obligation to ensure that regular times are dedicated to supervisee-focused discussions.

This process begins with an assessment of supervisee strengths and needs based upon the supervisee's completion of a self-assessment. Supervisors use self-assessments as a starting point to talk one-to-one with PARENTINGWELL staff about practice strengths and areas needing improvement. This discussion informs the development of an Individual Skill Development Plan (ISD) that, in turn, becomes the focus of subsequent monthly supervisee-focused discussions.

Other supervisory meetings during the month focus on client care, staff support, and/or administrative responsibilities. Of course, the supervisee's development plan is always relevant and provides context for how client focused and administrative supervision should be approached.

Purpose

A supervisory session that is focused on a practitioner's practice development allows supervisors to:

- Reflect with PARENTINGWELL staff about their practice.
- Mutually assess staff's knowledge, skills, and attitudes.
- Support staff in their practice development progression.

Outcomes

Consistent and meaningful supervision leads to:

- Supervisors have increased understanding of their staff's practice knowledge.
- Staff feeling more confident and supported in their work.
- Increase in staff's ability to apply practice skills.

 © ParentingWell 2019

- Reduced need for crisis-driven supervision
- Improved and consistent practice with a variety of children/families.

Frequency

Supervisory meetings that focus on staff's practice development, guided by the PARENTINGWELL Practice Profile self-assessment and ISD plan, should occur at least once each month. More frequent sessions could be scheduled based on staff knowledge and skill, an especially challenging workload, and/or concerns identified in clinical discussions or field observations.

Preparation

PARENTINGWELL staff should complete and give to their supervisor the self-assessment prior to the first supervisee-focused supervisory meeting. Supervisors ensure that this meeting is productive by reviewing the self-assessment and preparing questions prior to the meeting.

Conducting a Monthly Supervisee-focused meeting

It is important that supervisors:

- Protect the time needed, on a regular and predictable basis, to focus specifically on the development of the supervisee's skills, knowledge and attitudes. This models a commitment to caring for the welfare and development of the supervisee as well as the persons with whom the supervisee works.
- Create a learning environment and spend more time asking questions than giving direction in order to develop staff's critical thinking skills.
- Create a safe environment where staff can express emotions and respectfully disagree. A safe emotional environment allows staff to have difficult conversations with their supervisor. This, in turn, helps staff be better prepared to have them with families, referral sources, and other community providers.
- Schedule the meetings so there is plenty of time; don't rush.
- Collaboratively develop an ISD plan.

Tools

- Self-Assessment Work Sheet
- Individual Skill Plan

 © ParentingWell 2019
